# Neurophysiological Correlates of Musical and Prosodic Phrasing: Shared Processing Mechanisms and Effects of Musical Expertise

**DOI:** 10.1371/journal.pone.0155300

**Published:** 2016-05-18

**Authors:** Anastasia Glushko, Karsten Steinhauer, John DePriest, Stefan Koelsch

**Affiliations:** 1 Freie Universität Berlin, Berlin, Germany; 2 Integrated Program in Neuroscience, McGill University, Montreal, Quebec, Canada; 3 The Centre for Research on Brain, Language and Music (CRBLM), Montreal, Quebec, Canada; 4 School of Communication Sciences and Disorders, McGill University, Montreal, Quebec, Canada; 5 Program in Linguistics, Tulane University, New Orleans, Louisiana, United States of America; 6 Department of Biological and Medical Psychology, University in Bergen, Bergen, Norway; ARC Centre of Excellence in Cognition and its Disorders (CCD), AUSTRALIA

## Abstract

The processing of prosodic phrase boundaries in language is immediately reflected by a specific event-related potential component called the Closure Positive Shift (CPS). A component somewhat reminiscent of the CPS in language has also been reported for musical phrases (i.e., the so-called ‘music CPS’). However, in previous studies the quantification of the music-CPS as well as its morphology and timing differed substantially from the characteristics of the language-CPS. Therefore, the degree of correspondence between cognitive mechanisms of phrasing in music and in language has remained questionable. Here, we probed the shared nature of mechanisms underlying musical and prosodic phrasing by (1) investigating whether the music-CPS is present at phrase boundary positions where the language-CPS has been originally reported (i.e., at the *onset* of the pause between phrases), and (2) comparing the CPS in music and in language in non-musicians and professional musicians. For the first time, we report a positive shift at the *onset* of musical phrase boundaries that strongly resembles the language-CPS and argue that the *post-boundary* ‘music-CPS’ of previous studies may be an entirely distinct ERP component. Moreover, the language-CPS in musicians was found to be less prominent than in non-musicians, suggesting more efficient processing of prosodic phrases in language as a result of higher musical expertise.

## Introduction

The present study attempts to clarify a number of questions regarding the quantification and the functional significance of the *Closure Positive Shift* (CPS), a component in event-related brain potentials (ERPs) previously found to reflect boundary processing and phrasing in both language and music. One focus is on differences and similarities among musicians and non-musicians in these two cognitive domains. The second focus is on differences in how the CPS has typically been measured in language and music studies, as well as differences in their respective neurophysiological profiles. A main interest concerns the question of whether or not the CPS in language and music studies points to similar cognitive processes and can be viewed as support for a general mechanism underlying phrasing across domains.

### The CPS at prosodic boundaries in language

While the potential role of prosody in sentence processing was still quite controversial in 1996 (see special issue of the Journal of Psycholinguistic Research [1]), during the past twenty years prosodic phrasing has been shown to have an important and immediate impact on how we parse and interpret spoken utterances, with a particularly strong influence on syntactic parsing decisions (e.g., [2]). Segmentation of a speech signal into prosodic phrases is realized by listeners via a process that involves detecting particular acoustic cues that mark prosodic boundaries. Such cues include the prefinal lengthening of the last pre-boundary syllable, changes in pitch (especially boundary tones), and pause insertion (for German, see [3]). Prosodic cues differ cross-linguistically and additionally depend on the position of the prosodic boundary within the syntactic structure of the utterance [4]. The largest prosodic unit in the utterance has been referred to as the intonational phrase (IPh) and typically corresponds to syntactic phrases (for example, “*When a bear was approaching # the people started running away*”, where the IPh boundary is marked by the hash mark, ‘#’). IPh boundary processing is essential for syntactic parsing and is therefore crucial for language comprehension [2,5]. At the neurophysiological level, the processing of IPh boundaries in listeners is reflected by the Closure Positive Shift (CPS)–an ERP component seen at the offset of the pre-boundary phrase, often coinciding with the beginning of a pause [6]. The CPS is a positive waveform with a bilateral central scalp distribution near the midline and a duration of approximately 500 ms. To date, IPh boundary processing has been tested in similar ways in different languages, and CPS components have consistently been found in all studies comparing phrased and unphrased utterances (for reviews, see [7,8,9]). Similar but smaller CPS components have also been reported in silent reading (with one exception [10]), for instance at comma positions separating two clauses [11,12].

The prosodic CPS seems to be modulated by the strength of boundary markers in a graded manner (rather than being an all-or-none response), similar to other ERP components such as the N400 associated with lexical semantic processing [13] and the P600 reflecting structural processing difficulties in language [14] and music [15]. For example, it has been shown that boundaries with stronger pre-final lengthening and longer pauses elicit larger CPS amplitudes [16]. Boundaries that can be expected based on a previous context [17] or based on lexical information such as verb (in)transitivity [18] also seem to modulate the size of CPS components. In some cases, the CPS is preceded by a negativity at about 200 ms prior to the onset of the pause, resulting in a biphasic ERP response (e.g., [8]). This negativity is understood to be driven by early prosodic cues marking the phrase boundary, such as pitch variation and prefinal lengthening [7,8]. While an appropriate prosodic boundary can substantially facilitate sentence processing, syntactically incompatible boundaries often result in major misunderstandings later in the sentence (i.e., prosodically driven ‘garden-path’ effects) that elicit additional ERP responses such as N400 and P600 responses at the point of structural disambiguation ([6–8]; for analogous effects in second language learners, see [19]).

The CPS at prosodic boundaries has been demonstrated cross-linguistically in German [6], Dutch [7], English [8], Chinese [20], Korean [12], Japanese [21], Swedish [22], and French [23]. Importantly, its elicitation seems to be largely independent of lexical and syntactic information, given that it was observed even for delexicalized speech signals that did not contain any segmental information [24], and similarly for hummed sentence melodies [25]. Based on these findings, phrasing in language and other auditory domains may well rely on similar mechanisms, and the CPS was expected to reflect phrasing in musical stimuli as well [26]. Because not only intonational, but also intermediate prosodic phrase boundaries [16], as well as prosodic boundaries in delexicalized auditory signals [24] have been shown to elicit the CPS, it seems more appropriate to refer to this ERP component as reflecting ‘prosodic’ rather than ‘intonational’ phrasing.

### The post-boundary music-CPS

Similar to language, music is also organized in meaningful units of different lengths that guide our cognitive processing [27]. Musical phrases represent one of the levels in the generative hierarchical structure of music [28]. In Western tonal music, musical phrases are typically marked by prefinal lengthening (i.e., lengthening of the last note in the phrase) and subsequent short pauses. Moreover, harmonic, or musical syntactic, cues can be used to mark different types of phrase boundaries: A full cadence is a phrase-final sequence of tones or chords that represents strong syntactic closure marking the end of the entire period and does not imply further continuation (similar to a full stop in language; [29]). Other types of cadences (e.g., imperfect authentic cadences or half cadences) reflect weaker syntactic closures, often marking the end of a phrase but implying only a partial stop (similar to a comma in a sentence), after which the musical sequence may be continued.

In 2005, a seminal report on CPS-like positivities for musical phrase boundaries was published by Knösche and colleagues [30], using both electroencephalography (EEG) and magnetoencephalography (MEG) measures. Participants of that study were musicians, and the researchers found a positive ERP deflection in phrased melodies between 400 and 700 ms (peaking at around 550 ms) time-locked to the *onset of the first post-boundary note*. Similar to the language-CPS, this ERP effect occurred only in melodies with a phrase boundary and had a centro-parietal distribution near midline electrodes (hence the term ‘music-CPS’). The similarities between this *post-boundary* music-CPS and the language-CPS, along with the results of their MEG source localization (pointing to generators including the anterior cingulate cortex), brought the authors to conclude that the mechanisms underlying the CPS are not domain specific, and that the CPS may not reflect the detection of the phrase boundary but rather processes of attention and memory that “guide the attention focus from one phrase to the next” ([30], p. 259). Further investigation of the nature of the post-boundary music-CPS was undertaken by Neuhaus and colleagues who compared how musicians and non-musicians process musical phrase boundaries [31]. In line with Knösche and colleagues’ [30] findings, a centro-parietal positivity following boundaries was found in musicians. However, for the non-musicians the authors reported no post-boundary music-CPS but instead an early fronto-central negativity. The results were discussed as evidence for language-like processing of musical phrase boundaries in musicians, whereas non-musicians were thought to respond mostly to continuity expectancy violations. According to the authors, these new findings suggested that proficient boundary processing (as reflected by the CPS) may rely on a certain degree of expertise in the cognitive domain of interest. A number of follow-up studies challenging these conclusions will be discussed below.

### Methodological issues and differences between the language-CPS and the post-boundary music-CPS

Although reports on a post-boundary music-CPS seemed to confirm initial assumptions of a shared mechanism of phrasing in language and in music, a number of details resulted in skepticism regarding the equivalence of the language-CPS and the post-boundary music-CPS (e.g., [26]).

*First*, the electrophysiological profiles of language-CPS and post-boundary music-CPS differ in a number of important ways. Unlike the language-CPS found at the *onset* of speech boundaries, the music-CPS occurs much later (some 500 ms after the onset of the first *post*-boundary note), has a smaller amplitude, and a shorter duration (typically in the range of 200 ms, compared to about 500 ms during speech perception). The increased onset latency for this music-CPS may be partly accounted for by a larger degree of variability in music compared to language; that is, more context (and potentially a marker of the *end* of a pause) may be necessary in music to unambiguously identify a boundary. However, the latency difference between the language-CPS and the post-boundary music-CPS of almost 1000 ms is dramatic and may as well point to both different events eliciting these positivities and different mechanisms of phrasing. For example, the tones present during the first 600 ms after the onset of the post-boundary phrase also elicit enhanced onset P200s that are certainly not related to musical phrasing but to the larger acoustic contrast after a pause [24]. It is conceivable that the subsequent positivity (i.e., the post-boundary music-CPS) is related to these onset components (for details, see S4 Text). Importantly, music studies typically did not analyze ERPs elicited prior to the post-boundary note (i.e., during the pause), which is the time interval when the language-CPS is usually elicited (see also S2 Text). One exception is a report by Steinhauer, Nickels, Saini, and Duncan [26] describing some preliminary data on a music-CPS elicited earlier than it was reported by other studies of musical phrasing. The authors were the first to find a CPS during the pause (although still with slightly longer latencies than the language-CPS) for tone sequences lacking musical syntactic information characteristic of Western tonal music.

Another recent study worth mentioning is that by Silva and colleagues [32], who compared unphrased, well-formed phrased, and non-well-formed phrased melodic conditions. They reported a larger positivity time-locked to pause onset for well-formed phrased relative to non-well-formed conditions and, apparently, also relative to the unphrased condition, but–surprisingly–the latter contrast was excluded from further analysis. The positivity was interpreted as being similar to sentence-final wrap-up effects in language (an interpretation also offered for the language-CPS) ([33,34]; see also S3 Text). However, the series of pronounced auditory onset components (i.e., the P1-N1-P2 complex) associated with the notes “filling” the pause in unphrased melodies in this and other music studies render it virtually impossible to compare the ERPs to those of a condition with a pause between the phrases. Given that some authors have argued that the presence of a pause at musical phrase boundaries might be crucial for the elicitation of the post-boundary music-CPS [35] (and might, therefore, be essential for closure perception in music in general), in the present study we addressed this issue by including a condition in which the final note of the pre-boundary phrase is prolonged to the full duration of the pause in the phrased condition (thus eliciting no additional onset P200). Note, however, that while existing data [35] do suggest that the boundary pause is crucial for the perception of musical phrase boundaries (reflected in the post-boundary music-CPS), the question of whether the presence of the pause is necessary for the elicitation of the early ERP effects at the onset of the phrase boundary should be addressed by future studies.

The *second* concern arising from the previous music-CPS studies is that the absence of a post-boundary music-CPS in non-musicians and the conclusion that some expertise is necessary to elicit the component [31] seem somewhat counter-intuitive. Non-musicians can process musical features used in phrasing in music, such as certain timing cues [36], and music-syntactic regularities [37,38]). Moreover, current evidence indicates that children at age 3 show language-CPS components at intonational phrase boundaries [39] and that adult second language learners show CPS components in their second language right away, even at low levels of proficiency [19,23,40]. These notions question the requirement of special musical training in order to be able to process musical boundaries if the underlying neurocognitive mechanism of musical and prosodic phrasing is assumed to be the same. In line with these concerns, in a 2009 report by Steinhauer and colleagues [26], the CPS at boundary onset did not differ between musicians and non-musicians in either language or ‘music’. Note, however, that the ‘musical’ stimuli in that study were clearly different from Western tonal music. Moreover, the report [26] has been published as an abstract on preliminary analysis, with no detailed methods or results. Later that year, Nan, Knösche, and Friederici [41] then also reported a post-boundary music-CPS in *non*-musicians suggesting that the response may be task-dependent in this group of participants; however, that study still ignored the early period time-locked to the onset of the boundary pause.

It is possible that some differences between the CPS patterns in music and language may arise from the shortcomings related to specific baseline-correction procedures (e.g., with the baseline-correction interval being placed in the region where the stimuli in the compared conditions differ significantly) and certain stimuli characteristics used in the post-boundary music-CPS studies (e.g., the overlap of the music-CPS with the onset components reflecting auditory processing of the second post-boundary note). We address these issues in more detail in S4 Text.

### The present study

The current study aimed to investigate the neurophysiological correlates of phrase processing in music–and specifically, their similarities to those of prosodic phrasing in language. First, we examined whether a music-CPS similar to the language-CPS may be observed at the *onset* rather than the offset of the phrase boundary (a question that could not be addressed with the designs of previous music-CPS studies). Secondly, we also investigated the time interval of the previously reported post-boundary music-CPS in both musicians and non-musicians, addressing potential baseline issues and possible overlap of the music-CPS with onset components elicited by following notes (see also S2 and S4 Text). Finally, taking into account these methodological issues, we also aimed at replicating the findings of Neuhaus and colleagues [31] regarding the role of harmonic phrasing cues and the boundary strength in the appearance of the music-CPS. Unlike previous ERP studies of musical phrasing, the present study included an additional factor: stimulus familiarity/predictability (i.e., whether the musical phrase was presented for the first or the second time). This experimental paradigm was tested in both non-musicians and professional musicians, who participated in a standard language-CPS experiment as well. We used the typical language-CPS paradigm with the same groups of participants as well to compare the music data to the CPS for intonational phrase boundaries: the possibility exists that musicians differ from non-musicians even in the domain of prosodic phrasing due to transfer effects which are often reported for analogous processes in language and music (for review, see [42]).

## Methods

The ethics committee of the Faculty of Educational Sciences and Psychology at the Freie Universität Berlin approved the project (number of approval: 57/213). Written consent was obtained from each participant prior to the experiment.

### Participants

Thirty participants (14 musicians, 16 non-musicians) were recruited and tested for the present study. The group of professional musicians (nine females, five males) was recruited via distribution of flyers in two prestigious musical education institutions of Berlin (the University of Arts and the Academy of Music Hanns Eisler). Musicians had received a minimum of two years of training at one of these institutions; all played a musical instrument on a professional level (two participants specialized in singing), and all specialized in classical music. Non-musicians (six females, ten males) were recruited mostly via flyers distributed at the Free University of Berlin. Inclusion criteria for both groups were the following: absence of neurologic, psychiatric, or hearing deficits; normal or corrected vision; and German as a native language. Both musicians and non-musicians were paid for their participation.

The two groups were matched in age and IQ (for details see Table 1). IQ was assessed using the Multiple Choice Vocabulary Test, version B (*Mehrfachwahl-Wortschatz-B IQ Test*) [43] and a nonverbal strategic thinking test (part of the standard non-verbal IQ test battery–*Leistungsprüfsystem*) [44]. Only right-handed participants were included in the study (handedness was assessed using the Edinburgh Handedness Inventory [45]). An in-house musical expertise questionnaire including numerous questions regarding participants’ exposure to music (formal and informal, in terms of perception and production) and their general musicality was used as an additional measure for characterizing the two groups. Non-musicians had no more than one year of musical education, which had to have taken place at least five years before they participated in the experiment (14 out of 16 non-musicians did not have any formal musical training aside from the normal choir classes in primary school).

**Table 1:** Description of the experimental groups.

	Musicians		Non-musicians		Difference	
	Mean	SD^1^	Mean	SD^1^	*t* value	*p* value
Age (years)	28.75	3.30	25.43	8.43	1.45	.16
Verbal IQ^2^	29.5	3.61	28.56	3.65	-0.71	.49
Non-Verbal IQ^2^	32.43	4.43	30.25	3.47	-1.48	.15

^1^Standard deviation

^2^ In raw testing scores

### Design and materials

#### Language stimuli

The language stimuli were adapted for adult participants from the stimuli used by Pannekamp and colleagues [46] (modeled after [6]): some lexical units were changed and, therefore, new audio recordings of all sentences were made. The speech samples were produced by a female native German speaker who was familiar with the characteristics of the material.

Table 2 provides examples of the three types of sentences used. The first two (correct) sentences differed in the transitivity of the second verb, e.g., *grüßen* (*Engl*: 'to greet'; transitive), and *lächeln* (*Engl*: 'to smile'; intransitive). Because of the transitivity differences between these verbs, following German intonation patterns, an early intonational phrase boundary was present only in the Transitive condition (after the first verb, i.e., “*bittet*”), resulting in three IPhs in this type of sentences (see hash marks in Table 2, the word *“Tina”* is the direct object of the verb *“grüßen”*). In the Intransitive condition (where *“lächeln”* is intransitive, and *“Tina”* is the indirect object of the verb *“bittet”*), on the other hand, no early IPh boundary was present, resulting in two IPhs. The third type of sentences contained a prosody-syntax mismatch [6]. The Mismatch sentences were used to ensure that the stimulus materials recorded for this study allowed for natural language processing as indexed at minimum by adequate parsing of sentences in this condition. With respect to the examples in Table 2, these items were created by cross-splicing the first two types of sentences at the beginning of the affricate /ts/ in the word *zu*, such that the prosody until that point was drawn from the Transitive condition, while the remainder of the sentence following that point was intransitive. The early prosodic boundary prevents interpretation of *“Tina”* as the object of *“bittet”* and requires the parser to interpret it as the direct object at that verb *“lächeln”* (i.e., *to smile Tina), resulting in a syntactic violation [6,11]. At the position of the first boundary, the pause in the Transitive condition was between 550 and 600 ms in duration, while no phrase boundary was present at the same position in the Intransitive sentences. At the position of the second boundary, the pause in the Transitive condition was 200 ms in duration (to ensure that conditions did not differ significantly with respect to the length of the sentences), whereas in the case of the Intransitive condition it was 600 ms.

**Table 2:** Sample linguistic stimuli (sentences).

Condition	Example and English translation
Transitive	*Maxe bittet #*^1^ *Tina zu grüßen # und das Lied mitzusingen*. (Engl: 'Maxe asks to greet Tina and to sing a song.')
Intransitive	*Maxe bittet Tina zu lächeln # und das Lied mitzusingen*. (Engl: 'Maxe asks Tina to smile and to sing a song.')
Mismatch	*Maxe bittet # Tina zu lächeln # und das Lied mitzusingen. (Engl: *'Maxe asks to smile Tina and to sing a song.')

^1^ Intonational phrase boundaries (IPh) are marked with #.

Forty-eight sentences for each of the three conditions were created resulting in a total of 144 sentences. Each recorded sentence was between four and five seconds in length. The presentation of the stimuli was randomized separately for each participant.

#### Music stimuli

**Fig 1:**
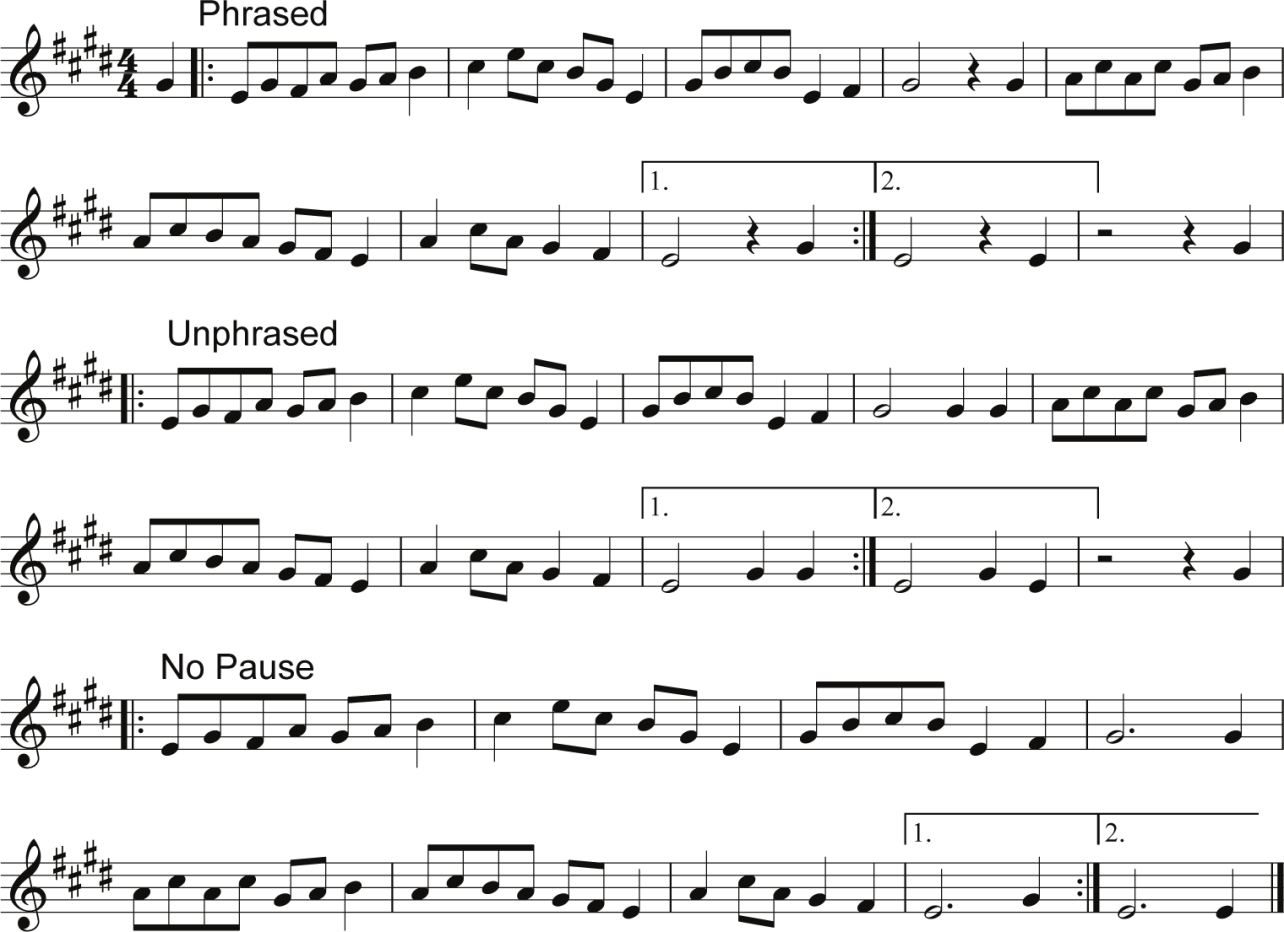
Notation of the sample music stimuli.

Twelve melodies were composed for this project following basic conventions of Western musical form. The monophonic music pieces were created as midi tracks in Sibelius First (Avid Technology, Inc., Burlington, USA) with a realistic acoustic piano sound. Each musical sample followed the same form, which was two four bar phrases creating an eight bar period, that was repeated (see Fig 1). A professional composer approved all music pieces. The total length of each track was 40.5 seconds.

The general structure of each melody comprised four musical phrases. The first four bars (the first phrase) ended with a weak musical syntactic closure: either ending on a fifth scale degree, as in half cadences, or on a third scale degree, as in imperfect authentic cadences (in Fig 1, the final note at the end of the first phrase is a G#). The bars four to eight (the second phrase) ended with a strong musical syntactic closure, represented by a tonic, typical for full cadences (in Fig 1, the second phrase terminates with an E, i.e., with the first scale degree). Further, these two phrases were repeated (forming the third and the forth phrase of the melody). The inclusion of the factor *Cadence* (weak vs. strong syntactic closure) was used to test the influence of musical syntactic cues on the ERP responses in the post-boundary music-CPS time window and between the phrases. The factor *Repetition* (the use of phrases presented for the first vs. for the second time) was included to investigate the effects of item familiarity/predictability on the CPS.

The final notes of each musical phrase were manipulated in their length and the presence of a pause between phrases to form three conditions that differed in the degree of boundary marking (i.e., the saliency of phrasing cues). Each melody was presented in all three conditions, which means that the music stimuli consisted of 36 melodies in total that were presented in a randomized order unique for each participant. These manipulations of degree of phrasing were consistent within a melody (i.e., all four boundaries in a melody belonged to the same conditions). The following three conditions were used (for notation, refer to Fig 1):

1. *Phrased*: at the phrase final positions, a half note (1200 ms long; in Fig 1, this note was a G# in the case of the weak syntactic closure, and E in the case of the strong syntactic closure) was followed by a quarter rest, indicating the last part of the phrase boundary. The first note after the boundary pause (in the example of Fig 1, it was always a G#) was either 300 or 600 ms long. The last note of each phrase (i.e., the half note) was slowly fading in amplitude in the next 600 ms (rather than being silenced sharply at the beginning of the pause) so that the melodies would be perceived as natural piano music pieces (see also S1 Audio);
2. *Unphrased*: at the phrase final positions, the half note was followed by either a quarter note, or two eighth notes (in place of pause in the Phrased condition; see also S2 Audio); and
3. *No Pause*: the phrase final notes were lengthened in order to fill in the pause (i.e., a 1800 ms long note was present at the end of the phrase boundary). This condition was used to investigate the ERP responses during the pause between the phrases, where the language-CPS is typically seen. Note that at the phrase boundary, significant differences between the sound intensity of Phrased and No Pause melodies appeared 500 ms prior to the offset of the pause. From the beginning of the post-boundary phrase, the two conditions were then again acoustically identical (see also S3 Audio).

### EEG recordings

The EEG was recorded from 32 Ag/AgCl electrodes (extended international 10 – 20 system) using the Brain Vision System (Brain Products GmbH, Munich, Germany) with a sampling rate of 500 Hz. Impedances were kept below 10 kΩ. Additional electrodes were attached at both mastoids (the right mastoid electrode was used as a reference), and a ground electrode was placed at the back of the participant’s neck. Four additional EOG electrodes were placed above and below the left eye (two channels), as well as laterally from each of the eyes (one channel per eye) to record vertical and horizontal eye movements.

### Procedure

First, both verbal and non-verbal IQ were assessed, as well as handedness, and musical expertise. Then, the EEG experiment was conducted. Two musical and two language stimuli served as a training block after which the experimental phase began. During the actual experiment, music and language stimuli were presented in separate blocks (counter-balanced across participants) of approximately 30 minutes in duration. Blocks were separated by a 5-minute-long break. During the presentation of each stimulus participants were instructed to fixate on a cross in the centre of a computer monitor. Following the presentation of each sentence, participants were presented with a question on the screen: *Wie natürlich fanden Sie den letzten Satz*? (*Engl*: 'How natural did you find the previous sentence?'). The corresponding prompt for the music stimuli was *Wie natürlich fanden Sie das letzte Musikstück*? (*Engl*: 'How natural did you find the last piece of music?'). Responses were provided using a 5-point scale from 'Completely Unnatural' to 'Completely Natural'. This task was used to maintain participants’ attention during the stimulus presentation and for comparison of behavioural ratings with ERP data. Participants were encouraged to blink during the question-answer period, and were instructed to avoid blinking during stimulus presentation. The entire experimental procedure lasted approximately 90 minutes.

### Data analysis

Recordings were analyzed using EEProbe (ANT, Enschede, The Netherlands). A band-pass filter from 0.3 to 30 Hz (FIR, 1001 points) was used to reduce muscle artifacts and remove slow drifts from the data. Trials contaminated with facial movements and other irregular artifacts were then eliminated by rejecting sampling points if they exceeded a 30 μV threshold (standard deviation in a 200 ms moving time window) at any channel. Eye movements were corrected using a regression-based statistical procedure implemented in EEProbe. No more than 35% of trials in the music part of the study and 17% in the language part were rejected due to artifacts in any condition (across subjects).

#### Language-CPS analysis

To quantify the language-CPS, ERP epochs were time-locked to the offset of the first verb (*“bittet”* in the example above) in the Transitive and Intransitive conditions (i.e., the onset of the pause in Transitive sentences; see Table 2) and lasted from −500 to 1000 ms. The time window for CPS analysis was 0 to 500 ms. Following previous language-CPS studies (e.g., [8]), two distinct baseline intervals were selected: (a) the 500 ms period preceding the offset of verb 1, and (b) the −50 to 50 ms interval relative to the offset of verb 1. As discussed in previous research (e.g., [24,30]), using multiple baselines in auditory studies can be crucial to the investigation of the robustness of effects.

#### Garden-path effects analysis

To study the garden-path effects resulting from the prosody-syntax mismatch (e.g., “*to smile Tina”), our ERP analysis contrasted the Intransitive and Mismatch items time-locked to the beginning of the second verb in each sentence using baseline-independent peak-to-peak measurement of the central and the posterior midline electrodes (i.e., Cz and Pz). A baseline-independent measure was used because prior to the trigger, the Mismatch condition differed prosodically from the Intransitive one (the sentence without an early boundary; see Table 2). To avoid artifacts related to prosodic differences between conditions, a peak-to-peak analysis was used, for which the data underwent 5 Hz low-pass filtering to avoid artifacts caused by noise-related peaks in any of the individual datasets (note that this additional filtering of the data was used only in the case of the garden-path effects analysis). The relevant time window for N400 amplitude minima was limited to 0 – 650 ms and for P600 maxima, it lasted from 600 to 1200 ms relative to the beginning of the second verb.

#### Music-CPS analysis

In the music stimuli, the analyzed ERPs were time-locked to the onset of the first note following the phrase boundary with the average time window lasting from −2000 to 1500 ms. We aimed at analyzing two time intervals.

The first time interval was, analogous to the language-CPS, the segment during the pause between the phrases in the Phrased condition (from 550 to 0 ms prior to the beginning of the first note of the second phrase). In this case, only Phrased and No Pause conditions were compared. As stated above, the contrast of Phrased and Unphrased conditions in this time interval would be impossible because the notes in the Unphrased condition would elicit auditory onset components absent in the Phrased melodies; see also S4 Text. Two baseline intervals were used (baseline_1: −1800 to −600 ms; and baseline_2: −2000 to −1800 ms). The main baseline_1 was placed immediately before the time window of interest, from the beginning until the end of the last pre-boundary note (−1800 to −600 ms). To control for possible effects of cadence differences, we compared results obtained using our main baseline_1 to those obtained using a more distant baseline_2 placed in the last 200 ms prior to the last note of the pre-boundary phrase (−2000 to −1800 ms time-locked to the end of the pause between the phrases). Unless the results acquired with the analyses using these baseline correction intervals differ, we describe the results of the analysis with the use of baseline_1.

Second, we compared ERP responses to Phrased, Unphrased, and No Pause conditions (similar to the study of Neuhaus and colleagues [31]) in the 450 – 600 ms time window (as it was done in the studies reporting the post-boundary music-CPS [41,47]). Visual inspection of the waveforms suggested that the ERP signals in the post-boundary music-CPS time window may plausibly have been a continuation of the effects in the prior time period (330 – 450 ms; where significant differences between Phrased and Unphrased conditions have already been reported in the literature [47]). We will hence refer to the ERP responses in the 330 – 600 ms time interval as the ‘post-boundary music-CPS’ (in contrast to the ‘boundary-onset music-CPS’, which we predicted to find earlier, in the −550 – 0 ms time window, i.e., at the onset of the pause, which corresponds to the onset of the phrase boundary). We separately analyzed items with 300 ms long post-boundary notes (for details regarding this analysis, see S4 Text) and those with 600 ms long post-boundary notes (nine melodies, four phrase boundaries each). This was done to investigate the effects of auditory onset components elicited by the second post-boundary note on the post-boundary music-CPS. In the case of long (600 ms) post-boundary notes, the post-boundary music-CPS time window should not be affected by the onset components elicited by the second post-boundary note (in contrast to previous post-boundary music-CPS studies; see also S2 and S4 Text). Therefore, if the post-boundary music-CPS was a product of pure differences in auditory onset components corresponding to the second post-boundary notes, we would see no differences between conditions when the long post-boundary notes were used. At the same time, when the first post-boundary note was exactly 300 ms long (comparable to the mean length of this note in previous post-boundary music-CPS studies, in which, however, the inter-trial differences in note lengths most likely caused latency jitter in the onset components for following notes), we would expect to see a typical phasic auditory onset ERP response in the post-boundary music-CPS time window (rather than a slow positive shift resembling the CPS in language; see S4 Text). In the investigation of items with long post-boundary notes, due to inconsistent results in the multiple baseline analyses (see S4 Text), we performed a baseline-independent investigation of the ERP responses in the 330 – 600 ms time window. Finally, we analyzed an earlier (but still post-pause) effect seen when strong and weak syntactic closure items were compared (i.e., investigation of the musical syntax effects; time window: 230 – 340 ms). Only results including relevant experimental factors and having emerged as statistically significant are reported.

#### Statistical analysis

Statistical analysis was computed using R software [48]. The data were analyzed with repeated measures ANOVAs using the “ez” package [49]. For the behavioural data analysis, we included Condition (Transitive vs. Intransitive vs. Mismatch for language stimuli, and Phrased vs. No Pause vs. Unphrased for music) as a within-subjects factor, and Group (Musicians vs. Non-Musicians) as between-subjects factor. For the ERP analysis, regions of interest were defined by assigning specific levels of the factors Laterality (Lateral vs. Medial), AntPost (Frontal vs. Central vs. Posterior), and Hemisphere (Left vs. Right) to each of the electrodes (see S2 Table). Midline and lateral electrodes were analyzed separately. For lateral electrodes, Condition (Transitive vs. Intransitive, and Transitive vs. Mismatch), Laterality (Lateral vs. Medial electrodes), Hemisphere (Right vs. Left) and AntPost (Anterior vs. Central vs. Posterior) were taken as within-subjects factors in the language-CPS analysis, while Group served as between-subjects factor. Only factors Group, Condition (Intransitive vs. Mismatch), and AntPost (Cz vs. Pz) were included in the analysis of garden-path effects. For the analysis of the music stimuli, we used Cadence (Strong vs. Weak syntactic closure), Pause (Phrased vs. No Pause vs. Unphrased), Repetition (First Playing vs. Second Playing), and Laterality, Hemisphere, and AntPost (see above) as within-subjects contrasts, and Group as between-subjects factor. For midline electrodes, analogous analyses were performed with the absence of the Laterality and Hemisphere factors. Follow-up analyses were carried out with the use of additional ANOVAs (when appropriate) and pairwise *t*-tests. Bonferroni-corrected *p*-values are reported in all cases of multiple comparisons. Moreover, in the case of violations of the sphericity assumption, Greenhouse-Geisser correction of *p*-values was applied.

## Results

### Behavioral data

**Fig 2:**
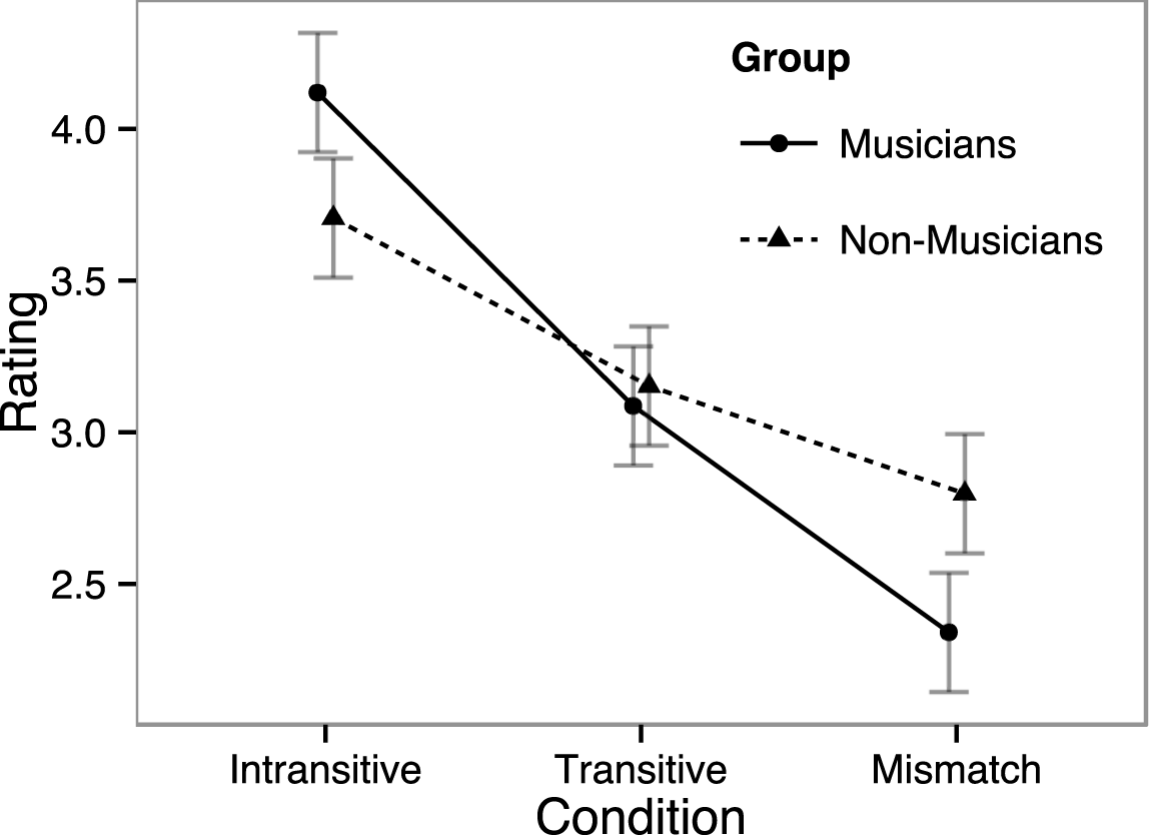
Naturalness ratings of sentences. Vertical bars indicate standard error of mean.

**Fig 3:**
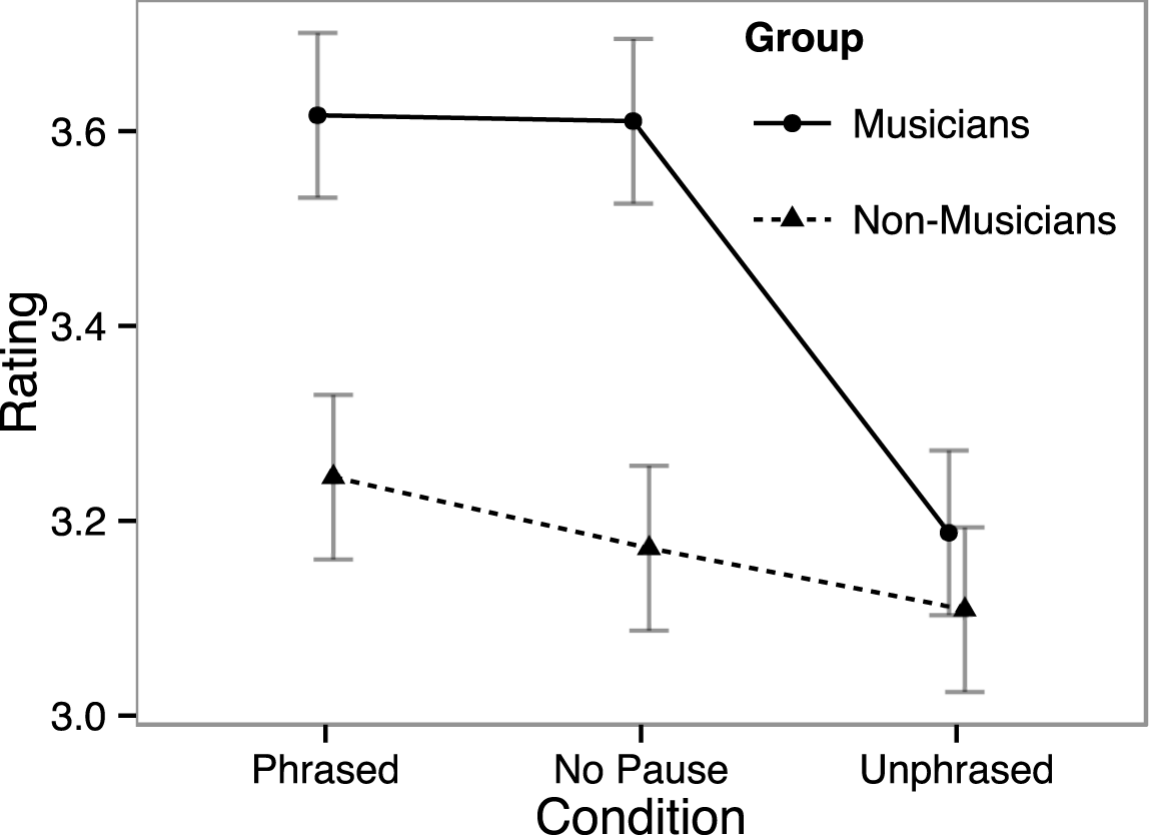
Naturalness ratings of melodies. Vertical bars indicate standard error of mean.

Figs 2 and 3 represent participants’ naturalness ratings of sentences per condition. The statistical analysis of sentence ratings revealed a significant main effect of Condition (*F* [2, 56] = 45.456, *p* < .001) and Group × Condition interaction (*F* [2, 56] = 4.927, *p* = .023). Post-hoc investigation of the effect of Condition showed that Intransitive items (i.e., sentences without an early phrase boundary) were rated as highest in naturalness, followed by the Transitive condition and then by the prosody-syntax Mismatch condition; all differences were significant (Intransitive vs. Transitive: *t* = 8.384, *p* < .001; Intransitive vs. Mismatch: *t* = 7.023, *p* < .001; Transitive vs. Mismatch: *t* = 3.636, *p* = .003). Follow-up analyses for the Group × Condition interaction within each group showed that the effect of Condition was significant for both musicians (*F* [2, 26] = 34.038, *p* < .001) and non-musicians (*F* [2, 30] = 13.215, *p* < .001). However, whereas musicians distinguished between all conditions (all *p*-values < .03), non-musicians did not rate the correct sentences as more natural than the prosody-syntax Mismatch condition (*t* = 2.115, *p* = .155; see Fig 2).

Consistent with the analysis of the sentence ratings, melody ratings (see Fig 3) yielded a significant main effect of Condition (*F* [2, 56] = 12.091, *p* < .001), as well as a Group × Condition interaction (*F* [2, 56] = 5.110, *p* = .012). The post-hoc pairwise analysis of the main effect of Condition revealed that the Unphrased condition was perceived as being slightly less natural than both Phrased (*t* = 4.629, *p* < .001) and No Pause items (*t* = 3.026, *p* = .015). However, the follow-up analysis of the interaction between Group × Condition showed that these effects were only present in the group of musicians (Condition: *F* [2, 26] = 13.699, *p* < .001; Phrased vs. Unphrased: *t* = 5.470, *p* < .001; No Pause vs. Unphrased: *t* = 3.767, *p* = .007).

### Language-CPS

**Fig 4:**
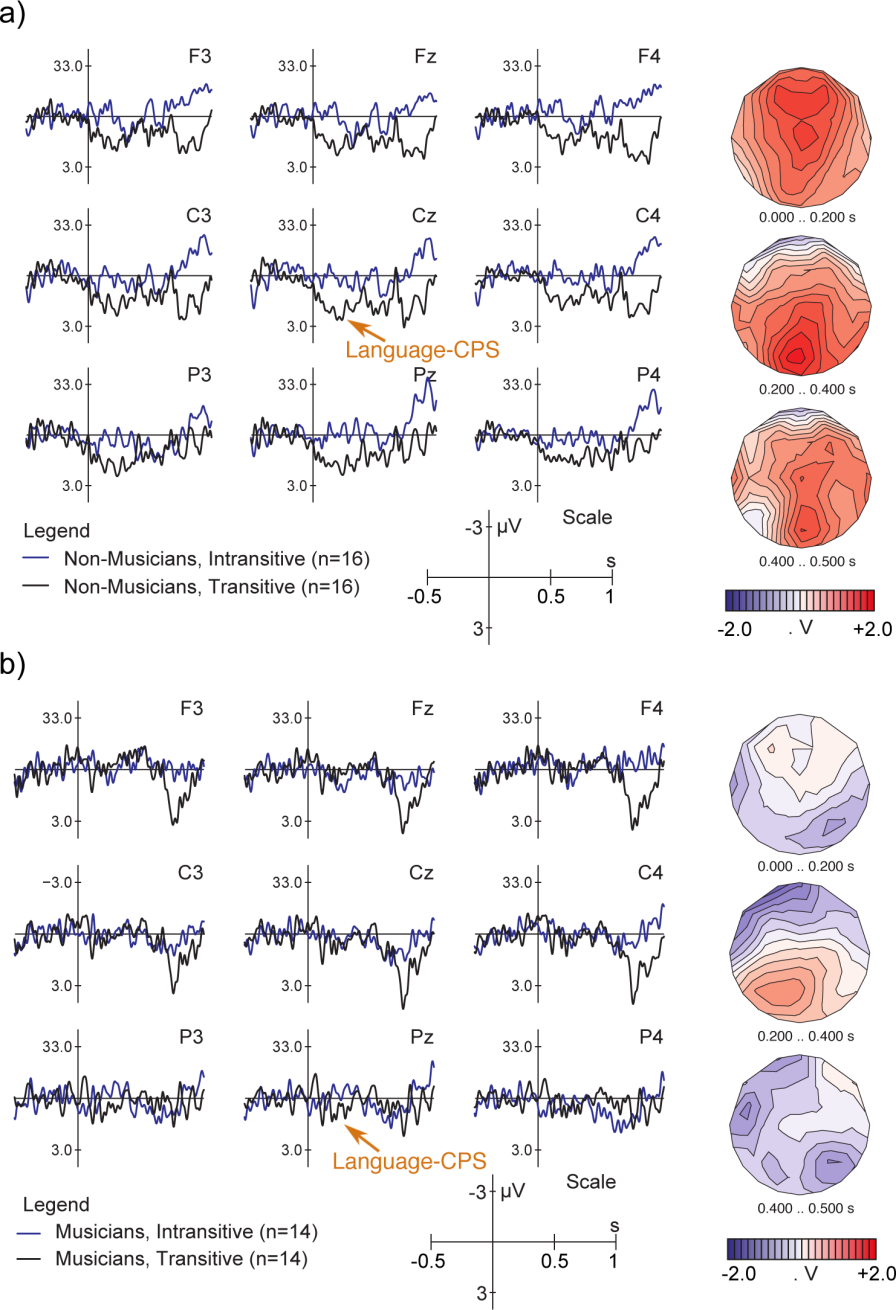
**ERP responses to IPh boundaries time-locked to the onset of the pause at the end of the IPh in (a) non-musicians and (b) musicians.** Baseline: −500 – 0 ms prior to pause onset. The CPS (closure positive shift) is a positive shift starting at approximately 0 ms in non-musicians and 200 ms in musicians and lasting for approximately 500 ms in non-musicians and 200 ms in the group of musicians. In musicians it was preceded by a short posterior negativity. Topographic maps represent the scalp distribution of the difference between two conditions in the specified time windows.

Fig 4A shows that in non-musicians, the beginning of the Pause in the Transitive condition (condition with a phrase boundary, see Table 2 for condition specifications) elicited a positive shift compared to the same segment in the ERP response to Intransitive sentences (condition without a phrase boundary). The positive shift was broadly distributed, with a bilateral posterior preponderance. The onset of this closure positive shift (CPS) seemed to be somewhat later, the duration slightly shorter, and the amplitude smaller in musicians (see Fig 4B) compared to non-musicians. The statistical analysis comparing Intransitive and Transitive sentences in the 0 to 500 ms time window, with the baseline set between −500 and 0 ms prior to the pause onset, yielded an interaction between Group and Condition when Intransitive and Transitive items were compared in the 0 to 500 ms time window (for midline electrodes: *F* [1, 28] = 6.820, *p* = .014; for lateral electrodes: *F* [1, 28] = 6.444, *p* = .017). This interaction reflected that the language-CPS was clearly present in non-musicians (midline: *F* [1, 15] = 18.381, *p* < .001; lateral: *F* [1, 15] = 18.058, *p* < .001) but not in musicians (midline: *F* [1, 13] < 1; lateral: *F* [1, 13] < 1). Independently of the Group factor, the main effect of Condition reached significance at midline electrodes (*F* [1, 28] = 7.522, *p* = .011), as well as at the medial electrode positions (Condition × Laterality: *F* [1, 28] = 5.145, *p* = .031; medial electrodes: *F* [1, 29] = 4.111, *p* = .052).

Notably, the Group × Condition interaction was not replicated in an analysis with the baseline set at −50 to 50 ms (midline: *F* [1, 28] < 1; lateral: *F* [1, 28] < 1). These inconsistencies were due to the negative peak present in the 0 – 50 ms time window in musicians, indicating that use of the −50 to 50 ms baseline was less appropriate than use of the standard −500 to 0 ms interval. To further clarify the difference between non-musicians and musicians in the language-CPS, a more fine-grained analysis of the data was performed on smaller, 100 ms long time windows using the standard −500 to 0 ms baseline. This analysis confirmed our initial observations. A relatively small and late CPS was significant in musicians only in the 200 – 400 ms time interval, while a typical CPS response was significant in all five 100 ms long time windows (0 – 100 ms, 100 – 200 ms, 200 – 300 ms, 300 – 400 ms, and 400 – 500 ms) in non-musicians (for details, see S3 Table and S5 Text).

### Garden-path effects

**Fig 5:**
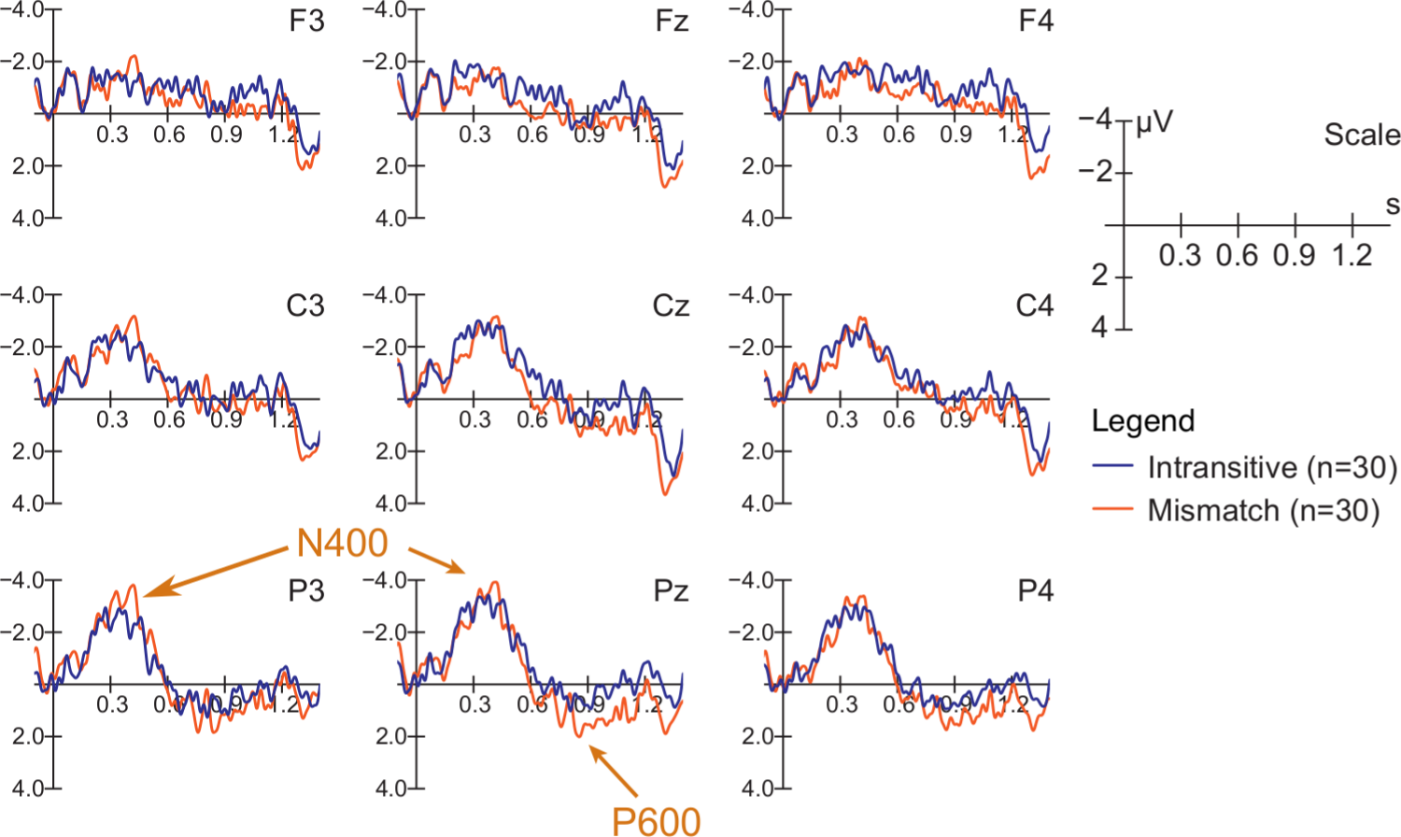
ERP responses to the sentences with prosody-syntax mismatch compared to intransitive items (without a phrase boundary). Baseline: −50 to 0 ms prior to the onset of the target verb. Note that statistical analysis of these data was performed based on the baseline-independent peak-to-peak analysis comparing the distance between the negative N400 and the positive P600 peaks (see Methods). The 5 Hz low-pass filter used to detect peaks was not used for producing this figure (for presentation purposes, we plotted the data filtered with a band-pass filter from 0.3 to 30 Hz, similar to the language-CPS data in Fig 4).

Fig 5 shows that although atypically small, a biphasic N400/P600 garden-path ERP pattern was replicated in the present study, revealing that the difference between N400 (a negative peak within 0 – 650 ms) and P600 (a positive peak within 600 – 1200 ms) peaks was larger for the Mismatch compared to the Intransitive condition (i.e., the condition without an early IPh) (*F* [1, 28] = 5.211, *p* = .039). The significant between AntPost × Condition interaction (*F* [1, 28] = 4.985, *p* = .034) reflected the finding that the effect was more pronounced at Pz (*F* [1, 28] = 6.725, *p* = .015). No Group × Condition interaction was found (*F* [1, 28] = 1.071, *p* = .31).

### Music-CPS

#### Boundary-onset music-CPS

**Fig 6:**
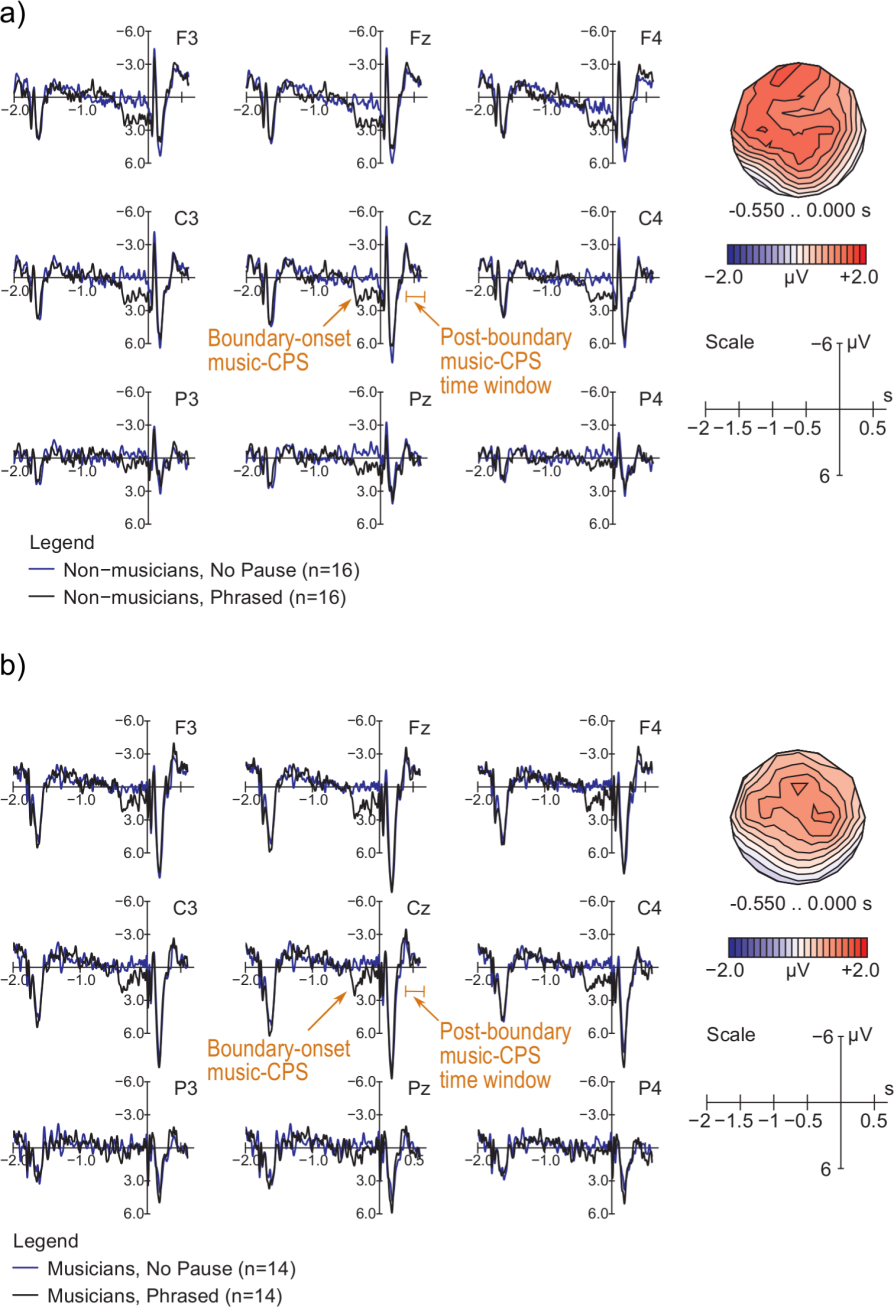
**ERP responses to musical phrase boundaries (Phrased vs. No Pause conditions) time-locked to the offset of the pause between the phrases in (a) non-musicians and (b) musicians.** Baseline: −1800 to −600 ms. The Pause between the phrases lasts from −550 to 0 ms, whereas the post-boundary music-CPS should be seen between 450 and 600 ms after the pause offset. To emphasize the temporal relationship between the ERP responses at boundary onset (i.e., during the pause) and the post-boundary music-CPS time window (elicited by the first post-boundary note), the latter one is also marked here. Topographic maps represent the scalp distribution of the difference between Phrased and No Pause conditions in the −550 to 0 ms time window.

Fig 6 shows the ERPs corresponding to the Phrased and No Pause conditions from -2000 to 600 ms, time-locked to the onset of the first post-boundary tone (vertical line at 0 ms). In this figure, ERPs after 0 ms reflect the processing of the post-boundary tone, whereas ERPs prior to 0 ms reflect the processing of earlier parts of the melody, including a pause in the Phrased condition that started around -550 ms. In both non-musicians (Fig 6A) and musicians (Fig 6B), the presence of the pause in the Phrased condition elicited a positive-going ERP wave between -500 and 0 ms compared to the control No Pause condition. This ERP effect at boundary onset strongly resembles previous findings of language-CPS components, in terms of both its latency (i.e., briefly after pause onset) and its duration (about 500 ms). As stated above, we will refer to this component as the ‘boundary-onset music-CPS’. The effect was distributed along the midline and was most prominent at frontal and central scalp areas (see isopotential maps in Fig 6). Statistical analysis yielded a main effect of Pause, reflecting the relative positivity of the ERP response to Phrased vs. No Pause items (midline: *F* [1, 28] = 26.623, *p* < .001; lateral: *F* [1, 28] = 12.785, *p* = .001). The effect was more prominent at the medial compared to lateral electrodes (Pause × Laterality: *F* [1, 28] = 10.165, *p* = .004; lateral: Phrased vs. No Pause: *F* [1, 28] = 5.115, *p* = .032; medial: Phrased vs. No Pause: *F* [1, 28] = 17.110, *p* < .001). Aside from the midline where the effect was seen at all electrodes, on lateral channels it was strongest over frontal and central scalp areas (AntPost × Pause: *F* [2, 56] = 30.033, *p* < .001; frontal: Phrased vs. No Pause: *F* [1, 28] = 23.514, *p* < .001; central: Phrased vs. No Pause: *F* [1, 28] = 23.138, *p* < .001). The AntPost × Pause × Laterality also reached significance, indicating that the distinction between the size of the Pause effect at the medial rather than lateral electrodes was most prominent at central electrodes (central, medial: Phrased vs. No Pause: *F* [1, 28] = 25.736, *p* < .001; central, lateral: Phrased vs. No Pause: *F* [1, 28] = 7.823, *p* = .009). All significant effects reported were also significant with the −2000 to −1800 ms baseline. Other statistically significant effects in this time window were related to musical repetitions, originated from differences that started much earlier, and likely reflected higher-level expectation processes that will be reported elsewhere [50]. Taking into account the structural similarity of the melodies used throughout the experiment, we performed an additional ‘split-half’ analysis of the data, comparing the first and second halves of the music part of the study. This analysis allowed us to investigate potential effects of boundary expectation that might have developed over the course of the experiment. No significant differences were observed when comparing the boundary-onset music-CPS in the first and second halves of the experiment.

#### Post-boundary music-CPS

**Fig 7:**
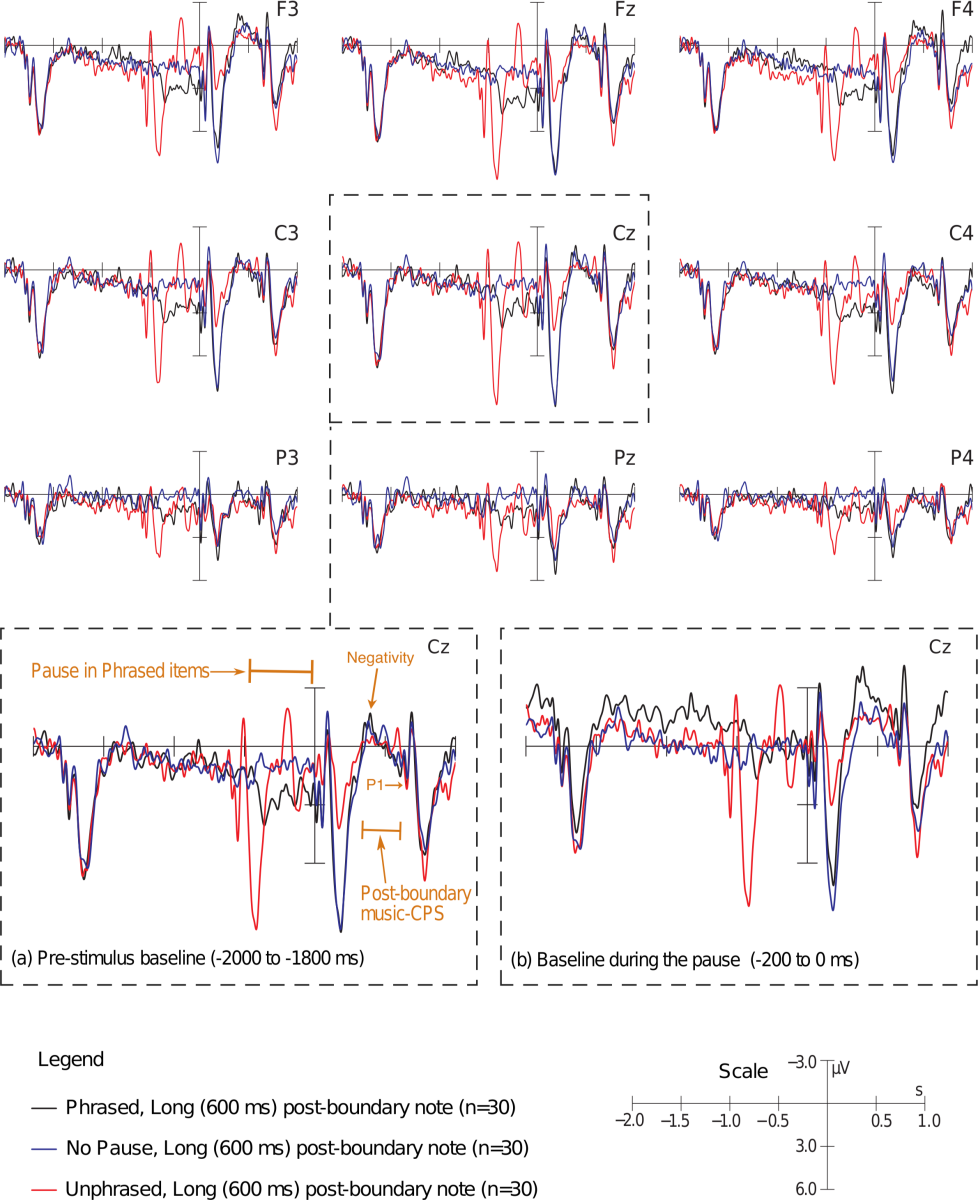
ERP effects of acoustical phrasing in music (i.e., presence of the pause and final lengthening). Only data from melodies with long (600 ms) first post-boundary notes are represented here. A pre-stimulus baseline (−2000 to −1800 ms) is used for the main plot of nine electrodes and the enlarged image of the Cz electrode (a). The enlarged Cz plot with the pre-stimulus baseline (a) is compared to the image of the same electrode (b) with the baseline placed during the Pause in the Phrased condition. The negative peak directly following the P2 elicited by the first post-boundary note is marked: it represents the start of the steep positive-going ERP slope in the Phrased condition. The slope ends at the auditory onset components elicited by the second post-boundary note (the onset P1 is marked).

Fig 7 shows the Pause effects in the post-boundary music-CPS time window (subsequent to the onset of the post-boundary phrase) for melodies in which the first post-boundary note lasted for 600 ms. There is no clear amplitude difference in this time interval that could be viewed as a replication of previous post-boundary music-CPS findings [30,31,41]. At the same time, it seemed that compared to Unphrased items, the ERP responses between 330 and 600 ms in the “more phrased” (Phrased and No Pause) conditions were characterized by a slightly steeper slope compared to Unphrased items (connecting the negative peak following the P200 [‘Negativity’ in Fig 7] and the onset P100 of the next tone). That is, we did not find a post-boundary music-CPS pattern resembling the one reported by either the previous music-CPS studies or the studies of language-CPS. This is in line with our hypothesis that auditory onset components elicited by the second post-boundary note in previous music-CPS studies could have produced a larger positivity (i.e., an onset P2 appearing at slightly different latencies across trials) in melodies with a boundary pause compared to those without a pause at phrase boundaries. Our analysis of melodies with 300 ms long post-boundary notes also supported this idea: we found the onset components for the second post-boundary note to be larger in Phrased compared to Unphrased items in musicians, whereas in neither of the groups did we see a slower centro-parietal positive shift in the data (see S4 Text).

The statistical analysis of items with long post-boundary notes confirmed our observations. No consistent significant amplitude differences were found with standard (baseline-dependent) analyses in the relevant post-boundary time intervals (see S1 Table). One might argue that the difficulty in qualifying and quantifying the post-boundary music-CPS is related to the fact that the ERP effect is at least partly influenced by the baseline choice (for details, see S4 Text). Therefore, and because we observed that the ERP responses in the 330 – 450 ms and the 450 – 600 ms time windows represent two parts of a monolithic shift in the “more phrased” conditions, we used an additional baseline-independent analysis that calculated the amplitude differences between the average ERP responses in these time windows within each condition and then compared these differences between conditions. We separately analyzed two different research questions, which in previous research were addressed independently as well: (1) whether there is an effect of musical phrasing (comparing Phrased and Unphrased conditions) [30]; (2) whether the music-CPS is modulated by acoustic boundary strength (including all three levels of the factor Pause into the statistical analysis) [31]. Regarding the first research question (i.e., Phrased and Unphrased comparison), the steepness of the slope (quantified via comparison of differences between mean amplitudes in the 330 – 450 ms and the 450 – 600 ms time windows) was higher for Phrased compared to Unphrased items at two midline electrodes (Pause × AntPost: *F* [2, 56] = 4.292, *p* = .029; Cz: Pause: *F* [1, 28] = 4.759, *p* = .038; Pz: Pause: *F* [1, 28] = 5.801, *p* = .023).

This effect (i.e., a steeper slope of the ERP for Phrased compared to Unphrased items) was also modulated by the type of cadential ending at the boundary: on lateral (non-midline) electrodes, it was seen for strong syntactic closures only: on medial frontal electrodes (AntPost × Pause × Cadence: *F* [2, 56] = 7.059, *p* = .005; strong syntactic closure, frontal electrodes: *F* [1, 28] = 3.575, *p* = .069; strong syntactic closure, frontal medial electrodes: *F* [1, 28] = 4.978, p = .034), as well as on medial central electrodes exclusively (AntPost × Laterality × Pause × Cadence: *F* [2, 56] = 4.152, *p* = .021; strong syntactic closure, central medial sites: *F* [1, 28] = 6.077, *p* = .020). Moreover, the ‘split-half’ analysis showed that the difference between Phrased and Unphrased items was more prominent in the second half of the musical phrasing experiment (midline: ExpPart × Pause: *F* [1, 28] = 5.277, *p* = .029; second part: Pause: *F* [1, 28] = 8.161, *p* = .008; lateral: Laterality × Pause × ExpPart: *F* [1, 28] = 7.330, *p* = .011; medial electrodes, second part: *F* [1, 28] = 6.184, *p* = .019).

The analysis of boundary strength including all three phrasedness conditions revealed only marginal effects, which could be attributed purely to the Pause (e.g., midline electrodes: Pause: *F* [2, 56] = 2.540, *p* = .088; lateral electrodes: Pause × AntPost: *F* [4, 112] = 2.140, *p* = .114; see also Table A in S4 Text). However, to qualify the effects of boundary strength, and because the boundary-onset music-CPS-like component (observed during the pause in the Phrased condition) was defined based on the comparison of Phrased and No Pause items, it was crucial to compare the difference in ERP responses between Phrased and No Pause melodies for differentiating the early and the late CPS components. Here, the Phrased condition had a larger shift than the No Pause condition (midline: *F* [1, 28] = 5.735, *p* = .024; lateral: *F* [1, 28] = 4.907, *p* = .035). In musicians, the effect was more right-lateralized than in non-musicians (Group × Laterality × Hemi × Pause: *F* [1, 28] = 4.909, *p* = .035; musicians, lateral electrodes, right hemisphere: Phrased vs. No Pause: *F* [1, 13] = 4.996, *p* = .044). Note that differences in post-boundary music-CPS lateralization between musicians and non-musicians have been previously reported when Phrased and Unphrased items were compared, but the pattern was reversed, with the CPS being more right-lateralized in non-musicians [41]. In non-musicians in our study the effect had a broad distribution.

While the effects evoked by acoustic boundary cues in the post-boundary music-CPS time window were, as stated above (see Phrased vs. Unphrased items contrast), modulated by the strength of the syntactic boundary cues, a further question is whether the post-boundary music-CPS can be driven by the syntactic boundary differences alone. However, we did not find clear indication of this being the case. The differences in the 330 – 450 and the 450 – 600 ms time windows originated from an earlier effect reminiscent of the P300 sub-component (see S6 Text). Note, however, that in the current experiment, both cadence and repetition effects may have been influenced by the fact that each melody was presented in three conditions (i.e., three times), which potentially caused their overlap with expectation effects.

## Discussion

### Musical expertise and prosodic phrasing

Professional musicians have often been used as a model for investigating brain plasticity due to musical training (for a review, see [51]). While both musicians and non-musicians were able to discriminate among the three language conditions, such that prosody-syntax mismatch received the lowest acceptability ratings, musicians were slightly more successful than non-musicians. A similar behavioral superiority for the group of trained musicians was also found in the music experiment, potentially pointing to a transfer effect across domains. It is worth mentioning, however, that in both groups the difference in acceptability between prosodically appropriate and mismatching sentences was less striking than in previous studies using very similar stimulus materials (e.g., [6]), whereas the difference between “correct” intransitives and “correct” transitives was larger in the present study. It is possible that the use of a graded rating scale (from 1 to 5) may have encouraged participants to focus on some subtle prosodic variations that were not part of the intended manipulations. On the other hand, the pause at the early closure in the Transitive condition was also relatively long, which might have additionally contributed to perception of these sentences as less natural than the Intransitive condition.

Turning to the ERP measures, the language experiment replicated the typical finding of a prosodic CPS in non-musicians [6–8,25,52]. As in previous studies, the language-CPS was most prominent at midline electrodes, started right after the onset of the pause, and lasted for several hundred milliseconds. When comparing these data to those of the trained musicians, we found that the profile of the language-CPS was significantly influenced by musical expertise. Musicians showed a later onset, shorter duration, and smaller amplitude of the CPS. This finding may seem somewhat surprising: Given that musicians were more successful in discriminating between the three sentence types (which required the integration of prosodic and lexical information), one might have expected a larger and more prominent CPS component in musicians. Alternatively, one could argue that the smaller CPS in musicians reflects a more efficient processing of the boundaries. For example, Kerkhofs, Vonk, Schriefers, and Chwilla [17] showed that sentences whose intonational phrase boundary was predictable given previous context elicited smaller CPS components at boundary positions compared to sentences with less predictable boundaries. If predictability results in more efficient processing, a reduced CPS amplitude in our group of musicians may be interpreted in the same way, recruiting fewer neural resources compared to non-musicians. This interpretation would be in line with previous findings of positive transfer effects from the music to the language domain in musicians (e.g., [53–54]). Note, however, that major inter-individual differences in ERP patterns were present *within* the group of musicians. Although our attempts at understanding the nature of this high inter-individual variability by using correlational analysis were not successful (see S5 Text), the absence of a homogenous ERP pattern in this group of participants suggests that there are factors other than the generic “musical expertise” that contribute to the modulation of the language-CPS. The question of which exact mechanisms (i.e., more effective low-level auditory processing or high level phrasing mechanisms) underlie more efficient processing of intonational phrases in musicians remains unresolved, and a possibility of individual predispositions influencing music and language skills must also be considered [55–56].

A central issue arising from the differences between musicians and non-musicians in intonational phrase processing is whether the mechanisms underlying musical and prosodic phrasing are completely or partially shared between these two domains. This will be discussed below when drawing parallels between ERP responses to musical phrasing and the language-CPS.

### Garden-path effects

The hypothesis regarding more efficient IPh processing in musicians is in line with the fact that both groups (musicians and non-musicians) showed the same ERP effects when processing garden-path structures in the Mismatch condition. Previous studies investigating the CPS and garden-path effects in a single experiment showed that when garden-path effects are present in the data, a CPS is always elicited at the phrase boundary if the same phrased and unphrased sentences but without prosody-syntax mismatch are compared [6,8]. That is, one can infer that the CPS, as a signature of IPh boundary detection, is necessary for the elicitation of the garden-path effects; and, therefore, if no differences were seen between musicians and non-musicians in garden-path effects (i.e., the detection of the prosody-syntax mismatch), both groups should have detected the IPh boundary. This provides support for the hypothesis of more efficient processing represented by the less prominent language-CPS in musicians compared to non-musicians.

Overall, the biphasic N400/P600 pattern reflecting garden-path effects for prosody-syntax mismatches was found to be less prominent than in previous studies (e.g., [8]). Whereas effects in the N400 time window have been rather small in some previous studies (e.g., Experiment 2 in [6]), the P600 was typically more prominent than the one observed in the present experiment [8]. We believe that the use of a 5-point scale in a “naturalness judgment task” might have contributed to reduced P600 effects in our data. In contrast to early reports on P600s and ‘syntactic positive shifts’ (e.g., [34,57]), the more recent literature has suggested that P600s cannot be viewed as monolithic components that exclusively reflect syntactic processing costs. Instead, a substantial part of the component’s amplitude seems to be task-related, reflecting a binary categorization of a sentence as either grammatical/acceptable or not (e.g, [58,59]).

### Musical phrasing

The results of the present study call into question the validity of previous findings of the post-boundary music-CPS ([30,47,41] and the effect in musicians in [31]), the appearance of which has been shown to be (1) potentially driven by the basic auditory processing of the second post-boundary note (reflected in the onset ERP components) and (2) at least partly dependent on the choice of baseline interval (see also S4 Text). In other words, we believe that in the previous studies of the post-boundary music-CPS, the presence of the positive ERP deflection in phrased melodies might be due to the combination of baseline-related differences and the pronounced auditory onset P2 component elicited by the second post-boundary note. The relatively long duration of this so-called music-CPS was likely due to the inter-stimulus variability in the length of the first post-boundary note (neither of the previous music-CPS studies kept the duration of the notes across trials constant). Such latency jitter might also explain the centro-parietal distribution of the post-boundary positive shift reported in previous studies: if the N1 and P2 onset components elicited in different trials at slightly different latencies overlapped in this time window, the fronto-central activities of these components might have at least partially cancelled each other out. In the current study, when we presented participants with melodies in which the onset components for the second post-boundary note fell into the post-boundary music-CPS time window but were elicited at constant latencies across trials, the respective ERP response was represented by a clear peak-like N1-P2 complex, very similar to the one elicited by the first post-boundary note (see S4 Text). At least in musicians in our study, this complex was more pronounced for Phrased compared to Unphrased melodies (likely due to habituation of the auditory onset components; see S4 Text). When these stimuli with first post-boundary notes of 300 ms in duration were used, in neither of the groups did we find a slower centro-parietal shift elicited in Phrased melodies that would resemble the language-CPS (see e.g., Fig B in S4 Text).

To overcome the confounding effects of the second post-boundary onset P2 component, in the current study we additionally used melodies in which the first post-boundary note was long enough (600 ms) to avoid the elicitation of any onset components in the time window of the post-boundary music-CPS. Once the contribution of these onset components was eliminated, it was not possible to detect the post-boundary music-CPS using the standard ERP analysis techniques employed in most language- and in all music-CPS studies so far. In a second step, therefore, we used a baseline-independent analysis measure. Yet even then, we found no main effect of phrasedeness when all three experimental conditions (Phrased, No Pause, and Unphrased) were included into the analysis. Only pairwise comparisons suggested that the Phrased condition had a somewhat steeper slope than the other two conditions between 330 and 600 ms after the onset of the post-boundary phrase (though even here the main effect reached significance on two electrodes only). Phrased items were characterized by a steep positive-going slope of the ERP wave starting at the *negative* peak following the first post-boundary onset P2 component (see e.g., Fig 7). This ERP slope was less prominent in the No Pause items and virtually absent in the Unphrased condition.

Because this effect originated from an initial negativity (in contrast to the positive shift reported by previous music-CPS studies) and was impossible to reliably quantify using standard baseline-dependent ERP measures employed in previous CPS research, we believe the effect was not related to phrasing but was rather elicited by a large auditory contrast between the pause and the beginning of the post-boundary phrase in the Phrased condition. Similar effects following the auditory onset P2 components and referred to as “sustained potentials” (SPs) have been consistently reported in studies of auditory tone perception (see Fig 1 in [60] as well as [61–64]). SPs are typically largest near the midline electrodes (e.g., [65]) and are pruned to habituation [60,63,64,66] (a feature also characteristic for the onset N1 and P2 components [67–69]). The latter quality of the SPs would explain the more pronounced response in conditions where the beginning of the post-boundary phrase is accompanied by a larger auditory contrast (i.e., from a pause to a new note). Our interpretation of these effects as being related to habituation is in line with the analysis of melodies with 300 ms long post-boundary notes, in which we believe that the slight differences in onset components between conditions were due to habituation as well (though in that case, for the second rather than the first note following the pause, these habituation effects were only present in musicians). The finding that the ERP effects in the post-boundary music-CPS time window were larger in the second part of the experiment and at boundaries with stronger syntactic closures is in line with the reports of the SPs being modulated by the level of attention paid to the stimuli by participants [64]. In other words, these responses seem to also be modulated by top-down processes (the specific nature of which is admittedly yet to be defined).

To sum up, we believe that the finding of the post-boundary music-CPS in the previous studies may be explained by the artifactual effects of baseline correction and by the differences in the auditory onset components elicited by the second post-boundary note. In the present study, when the potential methodological shortcomings of previous studies were addressed, we found no robust evidence for the post-boundary music-CPS evoked by neurocognitive mechanisms of phrasing. Instead, we interpret the ERP effects in the respective time window to be purely due to auditory contrast differences between experimental conditions. At the same time, however, we observed an earlier ERP component elicited in Phrased melodies–the boundary-onset music-CPS, which was quite similar to the CPS in our language experiment. Whereas previous studies on musical phrasing largely ignored the period during the pause (although this time window is most compatible with the one used in language studies on intonational phrasing), we specifically investigated it in our music experiment and discovered that Phrased melodies elicited a positive shift in relation to the No Pause items. Interestingly, this positive shift was elicited in both musicians and non-musicians and shared most characteristics of the language-CPS: it started soon after pause onset, had a considerable duration of some 500 ms, and–at least in the present study–had an amplitude comparable to that of the language-CPS as well. Given the physiological and functional similarities of the boundary-onset music-CPS and the language-CPS (for criteria used for ERP components qualification, see also [70]), we have reason to believe that in music, this boundary-onset positivity (and not the post-boundary music-CPS) is equivalent to the CPS components previously reported in language studies.

Several characteristics of the boundary-onset music-CPS require further clarification. First, the scalp distribution of this response was fronto-central, in contrast to the centro-posterior distribution of the language-CPS in our data. One potential reason for this might be related to the specific phrase boundary cues used in music and language. This interpretation is also supported by the fact that the fronto-central scalp distribution of the language-CPS has been reported by several studies [6,16,19], suggesting that differences in experimental materials might affect the scalp distribution of this ERP component. Second, auditory offset components (e.g., [64]) might have contributed to the responses in the boundary-onset music-CPS time window. However, the P2 in the complex of auditory offset ERP components is generally preceded by a negativity, either represented by a single N1 peak [61] or by two consecutive peaks [71–72]. In the present data, the boundary-onset music-CPS was not directly preceded by any seeming negativity. Moreover, whereas the offset P2 component is a clear peak quickly returning to the baseline level, in the present study, we observed a slow positive shift with a duration of at least 400 ms. In other words, if low-level auditory mechanisms contributed to the ERP responses in the boundary-onset music-CPS time window, they are likely to be complementary to the higher-level closure (grouping-related) processes.

Note that the quality of the syntactic cue at the boundary (strong versus weak syntactic closure) did not influence the appearance of the boundary-onset music-CPS. Similarly, in language, positive shifts have been observed for both intonational phrase boundaries at mid-sentence positions (i.e., the language-CPS [6]) and at sentence-final positions (e.g., [73]; for similar interpretations of sentence-final positivities, see [11]). However, the direct comparison of the mid-sentence language-CPS and sentence-final positive shifts has never been performed and is indeed worth an empirical investigation. With respect to the boundary-onset music-CPS, another potential direction for future research is to compare the characteristics of this ERP component in conditions that either do or do not provide musical syntactic phrase boundary cues (in addition to looking into different degrees of syntactic closure, as we did in the present study). The issue of how pre-final lengthening influences the boundary-onset CPS in music also warrants further investigation. Overall, the relative impact of all phrase boundary cues, including the pause, on this ERP response should be studied in the future—especially taking into account that the CPS in language studies has also been seen when the pause between phrases was omitted [6].

A final notable characteristic of the boundary-onset music-CPS concerns the fact that we found group differences in the language-CPS data but not in the boundary-onset music-CPS. These differences in the effects of musical expertise on the language-CPS and the CPS in music may be, again, due to the natural differences in boundary cues in language and music causing differences in the variability of ERP responses in the two domains. That is, the absence of group differences in the boundary-onset music-CPS component does not in and of itself provide disconfirming evidence for the hypothesis of shared neurocognitive mechanisms underlying this ERP response and the language-CPS.

## Conclusions

In the present study, we reported ERP data suggesting that musicians require less neurocognitive resources to process prosodic phrase boundaries in language (as reflected in the reduced language-CPS) compared to non-musicians. Moreover, we systematically investigated neurophysiological correlates of phrasing in music. After addressing the major methodological concerns arising from previous studies of the music-CPS, we found no evidence for the elicitation of the *post-boundary* positive shift resembling the language-CPS at musical phrase boundaries. The ERPs in the post-boundary music-CPS time window were instead likely influenced by lower-level auditory processing mechanisms unrelated to phrasing. At the same time, a robust positive shift was elicited by the onset of the phrase boundary (i.e., the offset of the pre-boundary phrase) in both musicians and non-musicians. This ERP component shared most characteristics of the language-CPS, and, therefore, presumably reflects closure of a grouped perceptual structure. The functional significance of this positive shift should be addressed in more detail by future research in order to establish to which extent the neurophysiological correlates of phrasing in music mirror those of prosodic phrasing in language.

## Supporting Information

S1 Audio Sample audio file representing the Phrased condition in the music part of the experiment. (MP3)

S2 Audio Sample audio file representing the Unphrased condition in the music part of the experiment. (MP3)

S3 Audio Sample audio file representing the No Pause condition in the music part of the experiment. (MP3)

S1 Table Results of the Global ANOVAs of neurophysiological correlates of music phrase boundary processing in the time period later to the beginning of the post-boundary phrase (time windows: 330 – 450 ms, 450 – 600 ms). (DOCX)

S2 Table Distribution of EEG channels in the regions of interests. (DOCX)

S3 Table Results of the Global ANOVAs of the language-CPS in the consecutive 100 ms long time windows time-locked to the onset of the boundary pause. (DOCX)

S1 Text The basic list of stimuli used in the language part of the study. Here, only Transitive and Intransitive conditions are represented. The Mismatch condition was built based on these two basic conditions (see Methods section).
(PDF)

S2 Text Details on CPS quantification. (DOCX)

S3 Text Methodological concerns arising from the study of Silva and colleagues (2014). (DOCX)

S4 Text Methodological concerns addressed by the present study. (DOCX)

S5 Text Investigating individual variability and its relation to the effects of musical expertise. (DOCX)

S6 Text Post-boundary cadence effects analysis. (DOCX)
