# Supplementary material for: Neurophysiological Correlates of Musical and Prosodic Phrasing: Shared Processing Mechanisms and Effects of Musical Expertise
Source: PLoS One. 2016 May 18;11(5):e0155300. doi: 10.1371/journal.pone.0155300 (PMC4871576; doi:10.1371/journal.pone.0155300)
Supplement: S1 Table — (DOCX) [file pone.0155300.s004.docx]

**S1 Table. Results of the Global ANOVAs of neurophysiological correlates of music phrase boundary processing in the time period later to the beginning of the post-boundary phrase (time windows: 330-450 ms, 450-600 ms). Only melodies with 600 ms long first post-boundary notes were included into this analysis.**

| **Baseline** | **Time Window** | **Electrodes** | **Effect^1^** | ***df*** | | ***F*** | ***p*** | |
| --- | --- | --- | --- | --- | --- | --- | --- | --- |
| -2000 to -1800 ms | 330 to 450 ms | Lateral | Group × AntPost × Cadence | 2 | 56 | 3.745 | .030 | |
|  |  |  | *AntPost × Rep^2^ × Pause* | 4 | 112 | 2.568 | .042 | |
|  |  |  | AntPost × Lat^3^ × Cadence | 2 | 56 | 3.354 | .042 | |
|  |  |  | *Group × AntPost × Rep × Pause* | 4 | 112 | 3.939 | .005 | |
|  |  |  | *Group × AntPost × Pause × Cadence* | 4 | 112 | 3.078 | .032 | |
|  |  |  | *Group × AntPost × Hemi^4^ × Rep × Pause* | 4 | 112 | 3.557 | .017 | |
|  |  | Midline | Group × AntPost × Cadence | 2 | 56 | 4.869 | 0.011 | |
|  |  |  | *AntPost × Pause × Rep* | 4 | 112 | 4.631 | 0.002 | |
|  | 450 to 600 ms | Lateral | Group × Rep | 1 | 28 | 5.686 | .024 | |
|  |  |  | AntPost × Cadence | 2 | 56 | 3.537 | .047 | |
|  |  |  | AntPost × Hemi × Cadence | 2 | 56 | 4.147 | .034 | |
|  |  |  | AntPost × Rep × Cadence | 2 | 56 | 3.885 | .040 | |
|  |  |  | *Group × AntPost × Hemi × Pause* | 4 | 112 | 2.711 | .034 | |
|  |  |  | *Group × AntPost × Rep × Pause* | 4 | 112 | 3.642 | .008 | |
|  |  |  | Group × AntPost × Hemi × Cadence | 2 | 56 | 4.648 | .024 | |
|  |  |  | *AntPost × Lat × Rep × Pause* | 4 | 112 | 2.756 | .031 | |
|  |  |  | *Group × AntPost × Hemi × Rep × Pause* | 4 | 112 | 3.263 | .028 | |
|  |  | Midline | Group × AntPost × Cadence | 2 | 56 | 3.343 | .043 | |
|  |  |  | *AntPost × Pause × Rep* | 4 | 112 | 3.563 | .009 | |
| -200 to 0 ms | 330 to 450 ms | Lateral | *Pause* | 2 | 56 | 7.798 | .001 | |
|  |  |  | Cadence | 1 | 28 | 9.328 | .005 | |
|  |  |  | *Lat × Pause* | 2 | 56 | 7.867 | .001 | |
|  |  |  | *Hemi × Pause* | 2 | 56 | 5.105 | .009 | |
|  |  |  | AntPost × Cadence | 2 | 56 | 3.269 | .045 | |
|  |  |  | *AntPost × Lat × Pause* | 4 | 112 | 5.006 | .001 | |
|  |  |  | *AntPost × Rep × Pause* | 4 | 112 | 3.576 | .009 | |
|  |  |  | *Hemi × Pause × Cadence* | 2 | 56 | 4.397 | .017 | |
|  |  |  | Group × AntPost × Hemi × Cadence | 2 | 56 | 3.709 | .044 | |
|  |  |  | Group × AntPost × Rep × Cadence | 2 | 56 | 3.826 | .046 | |
|  |  |  | *AntPost × Lat × Rep × Pause* | 4 | 112 | 4.798 | .001 | |
|  |  |  | *AntPost × Lat × Pause × Cadence* | 4 | 112 | 3.178 | .034 | |
|  |  |  | *Lat × Hemi × Pause × Cadence* | 2 | 56 | 3.330 | .043 | |
|  |  |  | *AntPost × Rep × Pause × Cadence* | 4 | 112 | 4.850 | .001 | |
|  |  | Midline | *Pause* | 2 | 56 | 10.290 | .001 | |
|  |  |  | Cadence | 1 | 28 | 5.582 | .025 | |
|  |  |  | Group × AntPost × Rep | 2 | 56 | 3.854 | .036 | |
|  |  |  | *AntPost × Pause × Cadence* | 4 | 112 | 6.107 | .001 | |
|  |  |  | *AntPost × Pause × Cadence × Rep* | 4 | 112 | 4.067 | .009 | |
|  |  |  | *AntPost × Pause* | 4 | 112 | 10.252 | < .001 | |
|  |  |  | *AntPost × Pause × Rep* | 4 | 112 | 9.774 | < .001 | |
|  | 450 to 600 ms | Lateral | Rep | 1 | 28 | 4.486 | .043 | |
|  |  |  | *Pause* | 2 | 56 | 4.770 | .019 | |
|  |  |  | Cadence | 1 | 28 | 8.122 | .008 | |
|  |  |  | AntPost × Cadence | 2 | 56 | 7.619 | .001 | |
|  |  |  | Lat × Cadence | 1 | 28 | 4.812 | .037 | |
|  |  |  | *AntPost × Lat × Pause* | 4 | 112 | 2.609 | .039 | |
|  |  |  | AntPost × Hemi × Cadence | 2 | 56 | 4.179 | .020 | |
|  |  |  | *AntPost × Pause × Cadence* | 4 | 112 | 3.526 | .009 | |
|  |  |  | *Hemi × Pause × Cadence* | 2 | 56 | 5.355 | .007 | |
|  |  |  | *Group × AntPost × Hemi × Pause* | 4 | 112 | 2.992 | .034 | |
|  |  |  | Group × AntPost × Hemi × Cadence | 2 | 56 | 6.981 | .002 | |
|  |  |  | *Group × AntPost × Pause × Cadence* | 4 | 112 | 2.526 | .045 | |
|  |  |  | *AntPost × Lat × Rep × Pause* | 4 | 112 | 2.598 | .040 | |
|  |  |  | *AntPost × Lat × Pause × Cadence* | 4 | 112 | 3.173 | .040 | |
|  |  |  | *Lat × Hemi × Pause × Cadence* | 2 | 56 | 3.585 | .034 | |
|  |  |  | *Group × AntPost × Rep × Pause × Cadence* | 4 | 112 | 3.706 | .007 | |
|  |  | Midline | *Pause* | 2 | 56 | 4.229 | .029 | |
|  |  |  | Cadence | 1 | 28 | 6.773 | .015 | |
|  |  |  | AntPost × Cadence | 2 | 56 | 5.805 | .005 | |
|  |  |  | *AntPost × Pause × Cadence* | 4 | 112 | 4.770 | .004 | |
|  |  |  | *Group × AntPost × Pause × Cadence × Rep* | 4 | 112 | 2.599 | .040 | |
|  |  |  | *AntPost × Pause* | 4 | 112 | 17.532 | < .001 | |
|  |  |  | *AntPost × Pause × Rep* | 4 | 112 | 7.605 | < .001 | |
| -2000 to 1000 ms | 330 to 450 ms | Lateral | Cadence | 1 | 28 | 6.310 | .018 | |
|  |  |  | AntPost × Cadence | 2 | 56 | 7.729 | .004 | |
|  |  |  | Lat × Cadence | 1 | 28 | 5.600 | .025 | |
|  |  |  | Group × AntPost × Cadence | 2 | 56 | 5.547 | .015 | |
|  |  |  | AntPost × Lat × Cadence | 2 | 56 | 6.479 | .005 | |
|  |  |  | *AntPost × Pause × Cadence* | 4 | 112 | 3.487 | .020 | |
|  |  |  | *AntPost × Rep × Pause × Cadence* | 4 | 112 | 3.007 | .039 | |
|  |  |  | *AntPost × Lat × Hemi × Rep × Pause × Cadence* | 4 | 112 | 2.510 | .046 | |
|  |  | Midline | Cadence | 1 | 28 | 6.303 | .018 | |
|  |  |  | AntPost × Cadence | 2 | 56 | 3.168 | .050 | |
|  |  |  | *AntPost × Pause × Cadence* | 4 | 112 | 3.110 | .018 | |
|  |  |  | *AntPost × Pause × Rep* | 4 | 112 | 5.521 | .002 | |
|  | 450 to 600 ms | Lateral | Cadence | 1 | 28 | 7.196 | | .012 |
|  |  |  | Lat × Rep | 1 | 28 | 5.925 | | .022 |
|  |  |  | Lat × Cadence | 1 | 28 | 12.138 | | .002 |
|  |  |  | Group × Lat × Rep | 1 | 28 | 4.324 | | .047 |
|  |  |  | AntPost × Hemi × Cadence | 2 | 56 | 5.268 | | .008 |
|  |  |  | *Group × AntPost × Hemi × Pause* | 4 | 112 | 5.404 | | .002 |
|  |  |  | Group × AntPost × Hemi × Cadence | 2 | 56 | 7.023 | | .002 |
|  |  |  | *Group × AntPost × Lat × Hemi × Pause* | 4 | 112 | 2.534 | | .044 |
|  |  |  | *Group × AntPost × Hemi × Rep × Pause* | 4 | 112 | 2.490 | | .047 |
|  |  |  | *Group × AntPost × Rep × Pause × Cadence* | 4 | 112 | 2.540 | | .044 |
|  |  | Midline | Cadence | 1 | 28 | 8.138 | | .008 |
|  |  |  | AntPost × Cadence | 2 | 56 | 5.939 | | .005 |
|  |  |  | AntPost × Rep | 2 | 56 | 3.767 | | .029 |
|  |  |  | *AntPost × Pause × Rep* | 4 | 112 | 4.625 | | .002 |
|  |  |  | AntPost × Cadence × Rep | 2 | 56 | 3.416 | | .049 |
|  |  |  | *Group × AntPost × Pause × Rep* | 4 | 112 | 2.634 | | .038 |
|  |  |  | *Group ×* *AntPost × Pause × Cadence × Rep* | 4 | 112 | 3.025 | | .033 |

^1^ Only statistically significant effects are reported; effects including the factor Pause are *italized*.

^2^ ‘Rep’ – Repetition

^3^ ‘Lat’ – Laterality

^4^ ‘Hemi’ - Hemisphere
