# Supplementary material for: Neurophysiological Correlates of Musical and Prosodic Phrasing: Shared Processing Mechanisms and Effects of Musical Expertise
Source: PLoS One. 2016 May 18;11(5):e0155300. doi: 10.1371/journal.pone.0155300 (PMC4871576; doi:10.1371/journal.pone.0155300)
Supplement: S2 Table — (DOCX) [file pone.0155300.s005.docx]

**S2 Table. Distribution of EEG channels in the regions of interests**

| Electrode | Category | Hemisphere | Laterality | AntPost |
| --- | --- | --- | --- | --- |
| Fz | midline | - | - | frontal |
| Cz | midline | - | - | central |
| Pz | midline | - | - | posterior |
| Fp1 | lateral | left | medial | frontal |
| Fp2 | lateral | right | medial | frontal |
| F7 | lateral | left | lateral | frontal |
| F3 | lateral | left | medial | frontal |
| F4 | lateral | right | medial | frontal |
| F8 | lateral | right | lateral | frontal |
| C3 | lateral | left | medial | central |
| C4 | lateral | right | medial | central |
| P3 | lateral | left | medial | posterior |
| P4 | lateral | right | medial | posterior |
| O1 | lateral | left | medial | posterior |
| O2 | lateral | right | medial | posterior |
| T7 | lateral | left | lateral | central |
| T8 | lateral | right | lateral | central |
| P7 | lateral | left | lateral | posterior |
| P8 | lateral | right | lateral | posterior |
| FC1 | lateral | left | medial | frontal |
| FC2 | lateral | right | medial | frontal |
| CP1 | lateral | left | medial | central |
| CP2 | lateral | right | medial | central |
| FC5 | lateral | left | lateral | frontal |
| FC6 | lateral | right | lateral | frontal |
| CP5 | lateral | left | lateral | central |
| CP6 | lateral | right | lateral | central |
