# Supplementary material for: Neurophysiological Correlates of Musical and Prosodic Phrasing: Shared Processing Mechanisms and Effects of Musical Expertise
Source: PLoS One. 2016 May 18;11(5):e0155300. doi: 10.1371/journal.pone.0155300 (PMC4871576; doi:10.1371/journal.pone.0155300)
Supplement: S3 Table — (DOCX) [file pone.0155300.s006.docx]

**S3 Table. Results of the Global ANOVAs and post-hoc comparisons of the language-CPS data (Intransitive vs. Transitive sentences) in the consecutive 100-ms-long time windows time-locked to the onset of the boundary pause. Baseline: − 500 – 0 ms.**

|  | Time window: 0 – 100 ms | | | |
| --- | --- | --- | --- | --- |
| *Lateral electrodes* | *df* | | *F^[[1]](#endnote-1)^* | *p* |
| Group × Cond^[[2]](#endnote-2)^ | 1 | 28 | 5.688 | .024 |
| Lat^[[3]](#endnote-3)^ × Cond | 1 | 28 | 5.671 | .024 |
| Group × AntPost × Lat × Hemi^[[4]](#endnote-4)^ × Cond | 2 | 56 | 3.458 | .038 |
| Post-hoc: Non-musicians, right medial frontal electrodes: Cond | 1 | 15 | 6.498 | .022 |
| *Midline electrodes* |  | | | |
| Group × Cond | 1 | 28 | 5.871 | .022 |
| Post-hoc: Non-musicians: Cond | 1 | 15 | 5.074 | .040 |
|  | Time window: 100 – 200 ms | | | |
| *Lateral electrodes* | *df* | | *F* | *p* |
| Cond | 1 | 28 | 8.488 | .007 |
| Lat × Cond | 1 | 28 | 11.506 | .002 |
| Post-hoc: Lateral electrodes: Cond | 1 | 28 | 5.397 | .028 |
| Post-hoc: Medial electrodes: Cond | 1 | 28 | 10.403 | .003 |
| AntPost × Lat × Hemi × Cond | 2 | 56 | 3.604 | .034 |
| Post-hoc: Right medial central electrodes: Cond | 1 | 28 | 11.660 | .002 |
| Post-hoc: Right medial frontal electrodes: Cond | 1 | 28 | 16.830 | < .001 |
| Post-hoc: Right lateral frontal electrodes: Cond | 1 | 28 | 14.972 | .001 |
| Group × AntPost × Lat × Hemi × Cond | 2 | 56 | 4.653 | .014 |
| Post-hoc: Non-musicians, left medial posterior electrodes: Cond | 1 | 15 | 7.016 | .018 |
| *Midline electrodes* |  | | | |
| Cond | 1 | 28 | 13.743 | .001 |
| Group × Cond | 1 | 28 | 5.139 | .031 |
| Post-hoc: Non-musicians: Cond | 1 | 15 | 17.254 | < .001 |
|  | Time window: 200 – 300 ms | | | |
| *Lateral electrodes* | *df* | | *F* | *p* |
| AntPost × Cond | 2 | 56 | 10.402 | .001 |
| Post-hoc: Posterior electrodes: Cond | 1 | 28 | 8.037 | .008 |
| Lat × Cond | 1 | 28 | 7.482 | .011 |
| AntPost × Hemi × Cond | 2 | 56 | 3.453 | .039 |
| Post-hoc: Left posterior electrodes: Cond | 1 | 28 | 9.743 | .004 |
| Post-hoc: Right posterior electrodes: Cond | 1 | 28 | 5.067 | .032 |
| *Midline electrodes* |  | | | |
| Cond | 1 | 28 | 4.815 | 0.037 |
| AntPost × Cond | 2 | 56 | 12.345 | < .001 |
| Post-hoc: Central: Cond | 1 | 28 | 4.586 | .041 |
| Post-hoc: Posterior: Cond | 1 | 28 | 9.922 | .004 |
|  | Time window: 300 – 400 ms | | | |
| *Lateral electrodes* | *df* | | *F* | *p* |
| Cond | 1 | 28 | 4.650 | .040 |
| AntPost × Cond | 2 | 56 | 8.536 | .004 |
| Post-hoc: Central electrodes: Cond | 1 | 28 | 6.687 | .015 |
| Post-hoc: Posterior electrodes: Cond | 1 | 28 | 12.664 | .001 |
| Group × AntPost × Hemi × Cond | 2 | 56 | 3.586 | .034 |
| *Midline electrodes* |  | | | |
| AntPost × Cond | 2 | 56 | 9.570 | .001 |
| Post-hoc: Posterior: Cond | 1 | 28 | 11.704 | .002 |
|  | Time window: 400 – 500 ms | | | |
| *Lateral electrodes* | *df* | | *F* | *p* |
| Group × Cond | 1 | 28 | 5.778 | .023 |
| Post-hoc: Non-musicians: Cond | 1 | 15 | 10.885 | .005 |
| Group × AntPost × Hemi × Cond | 2 | 56 | 6.804 | .002 |
| Post-hoc: Non-musicians, right posterior electrodes: Cond | 1 | 15 | 10.230 | .006 |
| *Midline electrodes* | *df* | | *F* | *p* |
| Group × Cond | 1 | 28 | 4.857 | .036 |
| Post-hoc: Non-musicians: Cond | 1 | 15 | 8.472 | .011 |

1. Only statistically significant results are reported. [↑](#endnote-ref-1)
2. ‘Cond’ – Condition [↑](#endnote-ref-2)
3. ‘Lat’ – Laterality [↑](#endnote-ref-3)
4. ‘Hemi’ – Hemisphere [↑](#endnote-ref-4)
