# Supplementary material for: Neurophysiological Correlates of Musical and Prosodic Phrasing: Shared Processing Mechanisms and Effects of Musical Expertise
Source: PLoS One. 2016 May 18;11(5):e0155300. doi: 10.1371/journal.pone.0155300 (PMC4871576; doi:10.1371/journal.pone.0155300)
Supplement: S1 Text — Here, only Transitive and Intransitive conditions are represented. The Mismatch condition was built based on these two basic conditions (see Methods section). (PDF) [file pone.0155300.s007.pdf]

S1 Text. The basic list of stimuli used in the language part of the study. Here, only Transitive and Intransitive conditions are represented. The Mismatch condition was built based on these two basic conditions (see Methods section).

01 Kevin verspricht Sophie zu schlafen und ganz lange lieb zu sein.
02 Kevin verspricht, Sophie zu küssen und ganz lange lieb zu sein.

03 Lena verspricht Sophie zu flitzen und Getränke zu kaufen.
04 Lena verspricht, Sophie zu helfen und Getränke zu kaufen.

05 Lena bittet Lukas zu tuten und lange rumzualbern.
06 Lena bittet, Lukas zu haschen und lange rumzualbern.

07 Sophie bittet Kevin zu kommen und das Spiel mitzumachen.
08 Sophie bittet, Kevin zu holen und das Spiel mitzumachen.

09 Lukas erlaubt Lena zu wüten und alles zu verraten.
10 Lukas erlaubt, Lena zu hauen und alles zu verraten.

11 Simba erlaubt Mietzi zu fauchen und die Vögel zu scheuchen.
12 Simba erlaubt, Mietzi zu baden und die Vögel zu scheuchen.

13 Lukas verbietet Kevin zu zappeln und vom Tisch aufzustehen.
14 Lukas verbietet, Kevin zu hänseln und vom Tisch aufzustehen.

15 Sophie verbietet Lukas zu nörgeln und ganz laut rumzuhupen.
16 Sophie verbietet, Lukas zu stören und ganz laut rumzuhupen.

17 Simba hilft Mietzi zu raufen und die Bälle zu schubsen.
18 Simba hilft, Mietzi zu ärgern und die Bälle zu schubsen.

19 Kevin hilft Lena zu lärmen und ganz laut zu klingeln.
20 Kevin hilft, Lena zu wecken und ganz laut zu klingeln.

21 Mietzi lehrt Simba zu rennen und die Katzen zu jagen.
22 Mietzi lehrt, Simba zu finden und die Katzen zu jagen.

23 Mietzi lehrt Simba zu schleichen und dabei aufzupassen.
24 Mietzi lehrt, Simba zu füttern und dabei aufzupassen.

25 Kevin verspricht Elke zu sitzen und am Tisch zu bleiben.
26 Kevin verspricht, Elke zu folgen und am Tisch zu bleiben.

27 Lena verspricht Elke zu saugen und das Geschirr zu spülen.
28 Lena verspricht, Elke zu stützen und das Geschirr zu spülen.

29 Elke bittet Kevin zu sausen und ganz schnell einzukaufen.
30 Elke bittet, Kevin zu suchen und ganz schnell einzukaufen.

31 Elke bittet Karsten zu tanken und eilig loszufahren.
32 Elke bittet, Karsten zu rufen und eilig loszufahren.

33 Karsten erlaubt Lena zu lachen und freudig loszukichern.
34 Karsten erlaubt, Lena zu kneifen und freudig loszukichern.

35 Mietzi erlaubt Simba zu schwimmen und den Kopf einzutauchen.
36 Mietzi erlaubt, Simba zu schnappen und den Kopf einzutauchen.

37 Simba verbietet Mietzi zu mauzen und die Krallen zu zeigen.
38 Simba verbietet, Mietzi zu strafen und die Krallen zu zeigen.

39 Simba verbietet Mietzi zu klettern und auf die Jagd zu gehen.
40 Simba verbietet, Mietzi zu locken und auf die Jagd zu gehen.

41 Kevin hilft Karsten zu malern und schön sauber zu machen.
42 Kevin hilft, Karsten zu kämmen und schön sauber zu machen.

43 Karsten hilft Lena zu puzzlen und das Rätsel zu lösen.
44 Karsten hilft, Lena zu kitzeln und das Rätsel zu lösen.

45 Lena lehrt Kevin zu rechnen und die Zahlen zu schreiben.
46 Lena lehrt, Kevin zu fragen und die Zahlen zu schreiben.

47 Mietzi lehrt Simba zu schnurren und die Ohren zu pflegen.
48 Mietzi lehrt, Simba zu kraulen und die Ohren zu pflegen.

49 Kevin verspricht Lena zu spucken und ganz schnell abzuhauen.
50 Kevin verspricht, Lena zu kratzen und ganz schnell abzuhauen.

51 Lena verspricht Kevin zu heulen und danach frech zu grinsen.
52 Lena verspricht, Kevin zu zwicken und danach frech zu grinsen.

53 Peter bittet Anna zu hopsen und ein Liedchen zu pfeifen.
54 Peter bittet, Anna zu loben und ein Liedchen zu pfeifen.

55 Anna bittet Peter zu klatschen und sehr laut rumzubrüllen.
56 Anna bittet, Peter zu schimpfen und sehr laut rumzubrüllen.

57 Anna erlaubt Lena zu quatschen und ein Liedchen zu trällern.
58 Anna erlaubt, Lena zu schminken und ein Liedchen zu trällern.

59 Stella erlaubt Bello zu kämpfen und dann lange zu schmusen.
60 Stella erlaubt, Bello zu kraulen und dann lange zu schmusen.

61 Stella verbietet Bello zu knurren und das Kind umzuwerfen.
62 Stella verbietet, Bello zu quälen und das Kind umzuwerfen.

63 Bello verbietet Stella zu jaulen und ganz laut rumzuwinseln.
64 Bello verbietet, Stella zu schubsen und ganz laut rumzuwinseln.

65 Lena hilft Kevin zu quengeln und ganz laut rumzuheulen.
66 Lena hilft, Kevin zu tadeln und ganz laut rumzuheulen.

67 Kevin hilft Peter zu toben und danach rumzukichern.
68 Kevin hilft, Peter zu knebeln und danach rumzukichern.

69 Peter lehrt Anna zu lügen und die Taschen zu stehlen.
70 Peter lehrt, Anna zu stossen und die Taschen zu stehlen.

71 Bello lehrt Stella zu schnüffeln und die Gefahr zu meiden.
72 Bello lehrt, Stella zu warnen und die Gefahr zu meiden.

73 Kevin verspricht Tina zu beten und ganz leise zu spielen.
74 Kevin verspricht, Tina zu mögen und ganz leise zu spielen.

75 Lena verspricht Maxe zu bleiben und die Erbsen zu essen.
76 Lena verspricht, Maxe zu knuddeln und die Erbsen zu essen.

77 Thomas bittet Kevin zu rülpsen und die andern zu stören.
78 Thomas bittet, Kevin zu ziepen und die andern zu stören.

79 Maxe bittet Tina zu lächeln und das Lied mitzusingen.
80 Maxe bittet, Tina zu grüssen und das Lied mitzusingen.

81 Gudrun erlaubt Thomas zu rutschen und sehr lange zu schaukeln.
82 Gudrun erlaubt, Thomas zu wiegen und sehr lange zu schaukeln.

83 Tina erlaubt Kevin zu schmatzen und danach loszulachen.
84 Tina erlaubt, Kevin zu pieksen und danach loszulachen.

85 Tina verbietet Lena zu jammern und den Hund anzuschreien.
86 Tina verbietet, Lena zu kneifen und den Hund anzuschreien.

87 Lena verbietet Thomas zu motzen und später wegzulaufen.
88 Lena verbietet, Thomas zu reizen und später wegzulaufen.

89 Maxe hilft Gudrun zu nageln und das Bild aufzuhängen.
90 Maxe hilft, Gudrun zu malen und das Bild aufzuhängen.

91 Kevin hilft Gudrun zu schummeln und alle auszulachen.
92 Kevin hilft, Gudrun zu necken und alle auszulachen.

93 Thomas lehrt Lena zu hocken und ganz schnell wegzuhüpfen.
94 Thomas lehrt, Lena zu retten und ganz schnell wegzuhüpfen.

95 Gudrun lehrt Maxe zu poltern und das Bad naßzuspritzen.
96 Gudrun lehrt, Maxe zu duschen und das Bad naßzuspritzen.
