# Supplementary material for: Neurophysiological Correlates of Musical and Prosodic Phrasing: Shared Processing Mechanisms and Effects of Musical Expertise
Source: PLoS One. 2016 May 18;11(5):e0155300. doi: 10.1371/journal.pone.0155300 (PMC4871576; doi:10.1371/journal.pone.0155300)
Supplement: S2 Text — (DOCX) [file pone.0155300.s008.docx]

**S2 Text. Details on CPS quantification**

Note that many early *speech studies* measured the CPS by simply comparing the ERPs of phrased and unphrased utterances across the entire sentence. This approach has the advantage of demonstrating that positive shifts in the phrased condition are, in fact, nicely aligned to the various boundary positions. However, Steinhauer [1] raised the issue that these analyses are not sufficient, because onset P200s of words following a phrase boundary are larger than those of co-articulated words in unphrased conditions, and that – due to variability in word and pause duration across trials – their averaged amplitudes may look like (and thus be mistaken for) a CPS. The only way of preventing this misinterpretation is to time-lock the ERP analysis to either pause onset or pause offset, where a real CPS can be distinguished from onset P200s (see also [2] and [1]). These concerns are not merely theoretical in nature. In a developmental study by Maennel and Friederici [3] that attempted to replicate Pannekamp and colleagues’ [4] finding of a CPS in even younger infants, the ERP analyses time-locked to sentence onset showed a CPS-like positivity near boundaries. Only their additional analyses time-locked to the post-boundary word revealed that these positivities were *exclusively* due to P200-like onset components. This finding also challenges Pannekamp et al.’s previous CPS finding in 6-month olds (whose data were analyzed only relative to sentence onset). Follow-up studies then confirmed that the CPS is not elicited until 3 years of age [5]. More recent speech studies have typically included CPS analyses time-locked to the boundaries and have consistently reported that the language-CPS starts just before the end of the pre-boundary phrase (and thus, during the pause and prior to the onset of the first post-boundary word). In contrast, *music-CPS studies* have generally focused on ERP components elicited after onset of the first *post*-boundary note, The ERP analyses were time-locked to the onset of the first post-boundary note, thereby avoiding ambiguities with averaged P200 components elicited by beginning of the post-boundary phrase.

**References**

1. Steinhauer K. Hirnphysiologische Korrelate prosodischer Satzverarbeitung bei gesprochener und geschriebener Sprache. (Neurophysiological correlates of prosodic sentence processing in spoken and written language) [dissertation]. Free University of Berlin. MPI Series in Cognitive Neuroscience 2001; 18.

2. Kerkhofs R, Vonk W, Schriefers H, Chwilla DJ. Discourse, syntax, and prosody: the brain reveals an immediate interaction. J Cogn Neurosci. 2007;19(9):1421–34.

3. Männel C, Friederici AD. Pauses and intonational phrasing: ERP studies in 5-month-old German infants and adults. J Cogn Neurosci. 2009;21(10):1988–2006.

4. Pannekamp A, Weber C, Friederici AD. Prosodic processing at the sentence level in infants. 2006;17(6):6–9.

5. Männel C, Schipke CS, Friederici AD. The role of pause as a prosodic boundary marker: language ERP studies in German 3- and 6-year-olds. Dev Cogn Neurosci. 2013;5:86–94.
