# Supplementary material for: Neurophysiological Correlates of Musical and Prosodic Phrasing: Shared Processing Mechanisms and Effects of Musical Expertise
Source: PLoS One. 2016 May 18;11(5):e0155300. doi: 10.1371/journal.pone.0155300 (PMC4871576; doi:10.1371/journal.pone.0155300)
Supplement: S3 Text — (DOCX) [file pone.0155300.s009.docx]

**S3 Text. Methodological concerns arising from the study of Silva and colleagues (2014)**

The effect (quantified between 500 and 700 ms) had an average amplitude of less than 1 microvolt (at Cz, the only electrode displayed) and was preceded by a much larger positivity (3−4 microvolts) between 150 and 300 ms in the unphrased condition that was found not to be significant; this either points to a low signal-to-noise ratio or problems in ERP quantification. This large positivity in the unphrased condition may very well be the onset P200 of the first note, as phrasing was prevented by replacing the pause with ‘filling tones’. Similarly, the (significant) positivity between 500 and 700 ms in the well-formed phrased condition may also be due to the average of onset P200s in that condition, since (a) the pause duration had an large standard deviation of 404 ms causing P200 latency variability and (b) the components’ fronto-central scalp distribution is that of a prototypical P200.

**References**

Silva S, Branco P, Barbosa F, Marques-Teixeira J, Petersson KM, Castro SL. Musical phrase boundaries, wrap-up and the closure positive shift. Brain Res. 2014;1585:99–107. doi: 10.1016/j.brainres.2014.08.025.
