# Supplementary material for: Neurophysiological Correlates of Musical and Prosodic Phrasing: Shared Processing Mechanisms and Effects of Musical Expertise
Source: PLoS One. 2016 May 18;11(5):e0155300. doi: 10.1371/journal.pone.0155300 (PMC4871576; doi:10.1371/journal.pone.0155300)
Supplement: S4 Text — (DOCX) [file pone.0155300.s010.docx]

**S4 Text. Methodological concerns addressed by the present study**

**The choice of the baseline correction interval**

One important issue to consider while investigating the post-boundary music-CPS is that because a positive shift also seems to accompany the pause between the phrases in phrased melodies (as it has been shown in the present study and as predicted by previous language-CPS research), the results of Nan and colleagues’ [1] study might have been affected by their primary choice of baseline (−200 to 0 ms time-locked to the onset of the post-boundary phrase, i.e., during the pause in the phrased condition). The proposal that baseline artifacts might be influencing the appearance of the post-boundary music-CPS is also supported by the inconsistency of N1 differences reported for the first post-boundary note across previous music-CPS studies (e.g., more pronounced N1 for unphrased conditions reported by Nan and colleagues in 2006 [2]; no differences in Knösche and co-authors in 2005 [3]; presence or absence of N1 differences dependent on the baseline choice in Neuhaus et al. [4]). Addressing these issues via the use of multiple baseline intervals is essential (e.g., see discussion in [4]); however, the nature of the differences in the ERP results as a function of the choice of baseline must also be explicitly investigated. An additional means of clarifying the status of results obtained with problematic baseline intervals involves using baseline-independent measures (where appropriate).

For the analysis of the post-boundary music-CPS in the present study, three different baseline correction intervals were used: −200 to 0 ms (corresponding to the pause in the Phrased condition), −2000 to −1800 ms (the last 200 ms during which all conditions were characterized by identical auditory signals), and −2000 to 1000 ms (a larger epoch including both pre- and post-CPS time windows). As stated in the main text of the current article, we saw distinct phrasedness-related ERP effects during the pause between the two musical phrases – specifically, a broadly distributed positivity in the Phrased condition elicited by the pause. This suggests that the direct baseline period within 200 ms prior to the onset of the post-boundary phrase (i.e., during the pause in the Phrased condition) might be confounded not only by the onset components of additional ‘filling notes’ in the Unphrased condition but also by a positive shift in phrased items - both of which render this baseline period inappropriate. Fig. 7 (b) shows that this was indeed the case in our study and, therefore, two alternative baseline choices were used (−2000 to −1800 ms and −2000 to 1000 ms).

A summary of effects that reached significance in the ANOVAs when different baselines were used in the analysis of items with long first post-boundary notes is provided in S1 Table. A *distant baseline* interval used by previous music-CPS studies (i.e., in our case between −2000 and −1800 ms) seems to have its weaknesses: it appeared that a long-lasting drift was causing divergence of the ERP responses to three experimental conditions; this drift was clearly seen at the auditory onset components elicited by the second post-boundary note. Our attempt to account for this drift using a −2000 to 1000 ms baseline seemed more appropriate but must be viewed as a compromise as it is conceptually different from a standard pre-stimulus baseline correction. Statistical analyses comparing averaged ERP responses in the two time windows between different conditions were in line with this observation. In the analyses with the −2000 and −1800 ms and the −2000 to 1000 ms baselines, no main effect of Pause was found; follow-up analyses of significant interactions led to a small number of significant effects, some of which were not consistent across analyses with different baselines (the data are available by request). These rather unreliable baseline-dependent results as well as the fact that both baseline correction intervals had their weaknesses motivated us to perform an additional baseline-independent analysis of the data (reported in the main text of the manuscript).

Yet even when baseline-independent analysis was performed, with the three levels of phrasedeness (Phrased, No Pause, and Unphrased) included into the ANOVA, we found no main effect of Pause. As seen from Table A, the effects resulting from a follow-up of significant interactions were almost exclusively seen on frontal electrodes (in contrast to the centro-parietal scalp distribution characteristic for the post-boundary music-CPS reported by previous studies). Moreover, we observed the difference between the conditions for only a subset of, but not for all phrases: i.e., exclusively for phrase boundaries with full cadences and for the non-repeated phrases (see Table A). The effects related to the degree of syntactic closure were in contrast to the findings by Neuhaus and colleagues [4] who found that the music-CPS (which was seen only in musicians) was elicited in both phrases with tonic and dominant endings and was frontal only in phrases ending on a dominant. In our study, the difference between Phrased and less phrased items (i.e., Unphrased and No Pause) was seen only in phrases ending on a tonic, and these effects were frontally distributed. This absence of the correspondence of our findings and the effects seen in previous studies led us to perform an additional analysis, where, like most previous music-CPS studies [1-4], we only included Phrased and Unphrased (but not No Pause) conditions into the ANOVA. This analysis is presented in the main text of the manuscript.

**Table A. Results of the global ANOVA and the pairwise follow-up analysis of the baseline-independent ERP measurements in the post-boundary music-CPS time window (330-600 ms). The ANOVA included all three levels of the factor Pause (Phrased, No Pause, and Unphrased). This analysis only involved melodies in which the first post-boundary note was 600 ms long.**

| **Electrodes** | **Global ANOVA: Effect^[[1]](#footnote-1)^** | ***F*** | ***df*** | | ***p*** |
| --- | --- | --- | --- | --- | --- |
| Lateral | AntPost *×* Repetition *×* Pause | 4.017 | 4 | 112 | 4.017 |
|  | AntPost *×* Pause *×* Cadence | 3.023 | 4 | 112 | 3.023 |
|  | AntPost *×* Laterality *×* Pause *×* Cadence | 2.842 | 4 | 112 | 2.842 |
|  | **Follow-up paired t-test: Effect** | ***t*** | ***df*** | | ***p*** |
|  | Frontal electrodes, first presentation:  Phrased > No Pause | 6.118 | 599 | | < .001 |
|  | Frontal electrodes, first presentation:  Unphrased > No Pause | 8.358 | 599 | | < .001 |
|  | Frontal electrodes, full cadence:  Phrased > No Pause | 6.941 | 599 | | < .001 |
|  | Frontal electrodes, full cadence:  Phrased > Unphrased | 5.197 | 599 | | < .001 |
|  | Medial frontal electrodes, full cadence:  Phrased > No Pause | 5.700 | 359 | | < .001 |
|  | Medial frontal electrodes, full cadence:  Phrased > Unphrased | 4.853 | 359 | | < .001 |
| Midline | **Global ANOVA: Effect** | ***F*** | ***df*** | | ***p*** |
|  | AntPost *×* Pause *×* Repetition | 3.515 | 4 | 112 | .032 |
|  | **Follow-up paired t-test: Effect** | ***t*** | ***df*** | | ***p*** |
|  | Cz, first presentation:  Phrased > No Pause | 2.831 | 59 | | .019 |
|  | Fz, first presentation:  Phrased > No Pause | 2.575 | 59 | | .038 |
|  | Fz, first presentation:  Unphrased > No Pause | 2.793 | 59 | | .021 |

**Potential overlap of the auditory onset components of either the preceding or the subsequent notes with the post-boundary music-CPS time window**

Another methodological concern arising from previous studies of the post-boundary music-CPS involves the fact that there is a possibility that the music-CPS *“originates from superimposed P2 components elicited by the following tones”* ([4], p. 489). This issue was addressed by the Neuhaus and colleagues via comparison of the scalp distributions of the onset components (typically frontal or fronto-central) and the post-boundary music-CPS (centro-parietal). However, the scalp distribution of the ERP components seen in the first 600 ms after the start of the post-boundary phrase can be influenced by the choice of the baseline interval used for averaging the EEG signals (e.g., [5]). Most importantly, potential inter-trial differences in the length of the first post-boundary note in the previous studies of the post-boundary music-CPS might have caused an overlap between N1 and P2 components associated with the second post-boundary note in the music-CPS time window. This overlap could have as well contributed to the centro-parietal scalp distribution of the post-boundary music-CPS: the frontal activations underlying both N1 and P2 might have at least partially cancelled each other out.

To test the hypothesis that auditory onset components elicited by the post-boundary notes contribute to the effects in the post-boundary music-CPS time window, we performed an additional analysis of a specific subset of our data. In previous studies investigating the music-CPS, where the length of the first note was stated, the average duration of the post-boundary notes was around 300 ms [1,2], which would place the P2 of the following note in the post-boundary music-CPS time window. Whereas other music-CPS studies did not report the precise length of the first post-boundary note, the length of notes and boundary pauses seemed to be in general characterized by pronounced inter-trial variability [3,4,6]. In the present study, we controlled for the length of the first post-boundary note via separately analyzing items with short and long first post-boundary notes (in all melodies used, the first post-boundary notes had a precise duration of either 300 or 600 ms, respectively).

As complementary evidence for the data from long (600 ms) post-boundary notes reported in the main text of the manuscript, here we report the analysis of items where the first post-boundary note was precisely 300-ms long (three melodies, three phrase boundaries each). Because baseline-dependent analysis revealed inconsistent results (similarly to the analysis of melodies with long post-boundary notes), and because the wave morphology of the effects in the post-boundary music-CPS time window resembled the typical waveform of onset ERP components, we performed peak-to-peak analysis comparing N1/P2 differences related to the onset of the second post-boundary note in Phrased and Unphrased items (contrasting conditions compared by the vast majority of previous studies [1,2,3,6]). We defined global peaks in the 340 – 450 ms time window (for the N1 minimum) and in the 420 – 550 ms epoch (for the P2 maximum). Only the first three phrase boundaries (out of four) in each melody were used in this analysis, since the second post-boundary note in the forth phrase was acoustically different from the analogous period in preceding phrases (by virtue of it being the last note in the melody). The factors Cadence and Repetition were not included in the ANOVA design. The analysis revealed that whereas the main effect of Condition (Phrased vs. Unphrased) did not reach significance, the Group × Condition interaction (midline: *F* [1, 27] = 8.689, *p* = .007) indicated that in musicians, *the peak-to-peak distance between N1 and P2 for the second post-boundary note in the Phrased condition was significantly larger than for the Unphrased melodies on midline electrodes* (*F*[1, 13] = 7.570, *p* = .016; see Figs. A and B).

To sum up, when the latencies of the notes in the stimulus materials were kept identical across trials, we found a slight difference between Phrased and Unphrased items on three midline electrodes in the group of musicians. The morphology of the ERP wave in this time window was indistinguishable from the waveform of auditory onset component in our study and did not resemble neither the post-boundary music-CPS found in previous studies (where the latency jitter in the onset of the second post-boundary note might have caused the broadening of the typically peak-like auditory onset components), nor the typical shape of the language-CPS (see e.g., Fig. 4 (a) for comparison).

Our data, therefore, suggest that some primary sensory processing differences between the conditions seem to affect the time windows of interest when the second note’s onset components fall into them. The slight differences between Phrased and Unphrased conditions in the post-boundary music-CPS window in such cases can be due to stronger habituation of the auditory onset components elicited by the second post-boundary note in the Unphrased condition compared to the less pronounced habituation in the Phrased condition, in which the post-boundary phrase was preceded by a period of absolute silence (for analogous habituation effects see, for example, [7]). Whereas in our study, the differences between the onset components elicited by the second post-boundary note in the Phrased and Unphrased conditions were rather minimal (and seen only in one group of participants), it is crucial to point out that if the investigation of the post-boundary music-CPS time window is performed using baseline-dependent analysis measures, the size of the effect might differ drastically due to confounding effects of baseline correction.

**Fig. A. Peak-to-peak N1/P2 distances elicited by the second post-boundary note in professional musicians for items with a short first post-boundary note.** Only data from midline electrodes are represented on the plot. The bold horizontal lines outline the means per condition; the boxes represent the inter-quartile range; the lower and the upper whiskers mark the lowest and the highest value within the 1.5 × inter-quartile range from the closest box boundary; the dots represent outliers.

**Fig. B. ERP effects of acoustical phrasing in music (i.e., presence of the pause) in the group of musicians.** Only data from melodies with short (200 ms) first post-boundary notes are represented here. Onset P2 components for the first, the second, and the third post-boundary notes are marked. The baseline is placed between 250 and 300 ms after the onset of the first post-boundary note for presentation purposes only. Only midline electrodes are plotted. Because it was impossible to define an appropriate baseline in the investigation of the post-boundary music-CPS time window in our study, it is difficult to conclude to which extent the N1 and P2 components contributed to the increased N1/P2 peak-to-peak distance in the Phrased condition compared to Unphrased items in musicians.

Overall, taking into account the methodological concerns arising from previous studies, we believe that the combination of baseline-related effects and the onset components of the post-boundary notes might have caused the effects seen in the post-boundary music-CPS time window in previous studies. When these methodological issues were addressed, we saw no evidence of a positive shift resembling the language-CPS in the post-boundary music-CPS time window. We warn future studies that the length of the first post-boundary note (as well as the inter-trial variability in this length) should be taken into account when the goal of investigation is to disentangle higher-level phrasing processing from lower-level auditory change detection in the post-boundary music-CPS time window. At the same time, a separate problem concerns the fact that auditory onset components elicited by the first post-boundary phrase are strikingly different in Phrased and Unphrased conditions, making it not only impossible to choose an appropriate baseline correction interval but also *rendering any effects, including the baseline-independent ones, difficult to interpret as the possibility exists that the ERP signal merely reflects the return of the neurophysiological responses to some baseline level*. It seems crucial to develop experimental paradigms that would control for this kind of confound but since the timing of the “post-boundary music-CPS” is tight to the auditory changes at the phrasal boundary, no simple solution is on the table.

**References**

1. Nan Y, Knösche TR, Friederici AD. Non-musicians’ perception of phrase boundaries in music: A cross-cultural ERP study. Biol Psychol [Internet]. 2009 Sep;82(1):70–81. doi:10.1016/j.biopsycho.2009.06.002.

2. Nan Y, Knösche TR, Friederici AD. The perception of musical phrase structure: a cross-cultural ERP study. Brain Res [Internet]. 2006 Jun;1094(1):179–91. doi:10.1016/j.brainres.2006.03.115.

3. Knösche TR, Neuhaus C, Haueisen J, Alter K, Maess B, Witte OW, et al. Perception of phrase structure in music. Hum Brain Mapp [Internet]. 2005;24(4):259–73. doi:10.1002/hbm.20088.

4. Neuhaus C, Knösche TR, Friederici AD. Effects of musical expertise and boundary markers on phrase perception in music. J Cogn Neurosci [Internet]. 2006 Mar;18(3):472–93. doi:10.1162/089892906775990642.

5. Steinhauer K, Drury JE. Brain & Language On the early left-anterior negativity (ELAN ) in syntax studies. Brain Lang. 2012 Feb;120(2):135–62. doi:10.1016/j.bandl.2011.07.001.

6. Silva S, Branco P, Barbosa F, Marques-Teixeira J, Petersson KM, Castro SL. Musical phrase boundaries, wrap-up and the closure positive shift. Brain Res. 2014;1585:99–107. doi:10.1016/j.brainres.2014.08.025.

7. Fruhstorfer H. Habituation and dishabituation of the human vertex response. Electroencephalography and clinical neurophysiology. 1971 Apr 1;30(4):306-312. doi:10.1016/0013-4694(71)90113-1.

1. Only statistically significant effects are reported throughout the table. [↑](#footnote-ref-1)
