# Supplementary material for: Neurophysiological Correlates of Musical and Prosodic Phrasing: Shared Processing Mechanisms and Effects of Musical Expertise
Source: PLoS One. 2016 May 18;11(5):e0155300. doi: 10.1371/journal.pone.0155300 (PMC4871576; doi:10.1371/journal.pone.0155300)
Supplement: S5 Text — (DOCX) [file pone.0155300.s011.docx]

**S5 Text. Investigating individual variability and its relation to the effects of musical expertise**

**Language-CPS**

Visual inspection of single subject averages showed that there is considerably greater inter-individual variability in the patterns of the ERP response time-locked to the pause onset within the group of musicians (only six of them had a clear CPS), whereas 14 out of 16 non-musicians showed a qualitatively distinguishable CPS-like pattern. To investigate this inter-individual variability, similarly to Tanner and Van Hell [1], we computed the difference between the means for each individual for a generic centroparietal region of interest (including Cz, Pz, CP1, CP2, P3, P4) for the 0 – 500 ms time window and ran bivariate correlations between these values and relevant factors such as sentence ratings and the various scales of the musicality questionnaire (i.e., self-perception as musical, number of music practice hours per week, years of music education, starting age of music education, hours of active music listening per day). None of the variables was a significant predictor of language-CPS amplitude, neither in the smaller time window targeting the potentially delayed and shortened language-CPS in musicians (84 to 400 ms; defined based on visual inspection of the averages) or in the generic 0 to 500 ms time window, regardless of whether all participants or only musicians were included.

**Music- and language-CPS**

To investigate the potential similarities between music and language-CPS, we performed correlational analysis aiming at following-up on the relationship between (1) the CPS in language and the boundary-onset music-CPS, (2) the CPS in language and the post-boundary music-CPS, and finally (3) the two music-CPS components. None of the correlations reached significance. Possible explanations for not having found any significant correlations between the sizes of the ERP effects can be traced to the lack of inter-individual variability in the music-CPS data and/or the fact that using correlations between average responses in pre-defined time windows is an imprecise measure of establishing whether two ERP components are related (but see also Discussion section of the manuscript). Note that neither our attempts of correlational analysis of the language-CPS data with certain behavioural measures, nor several previous studies using analogous approaches [1] have found predicted relationships in the ERP data. Large-scale studies and/or more fine-graned statistical approaches (e.g., general additive modelling; for an example, see [2]) might shed light on the potentially multifactorial relationships that exist between the CPS in music, and language, and factors related to music expertise.

**References**

1. Tanner D, Van Hell JG. ERPs reveal individual differences in morphosyntactic processing. Neuropsychologia [Internet]. 2014 Apr;56:289–301. doi:10.1016/j.neuropsychologia.2014.02.002.

2. Meulman N, Wieling M, Sprenger SA, Stowe LA & Schmid MS. Age effects in L2 grammar processing as revealed by ERPs and how (not) to study them. Poster presented at the Annual Meeting of the Society for the Neurobiology of Language; Amsterdam; 2014 Aug 27-29.
