# Supplementary material for: Neurophysiological Correlates of Musical and Prosodic Phrasing: Shared Processing Mechanisms and Effects of Musical Expertise
Source: PLoS One. 2016 May 18;11(5):e0155300. doi: 10.1371/journal.pone.0155300 (PMC4871576; doi:10.1371/journal.pone.0155300)
Supplement: S6 Text — (DOCX) [file pone.0155300.s012.docx]

**S6 Text. Post-boundary cadence effects analysis**

**Results**

At the falling end of P2 elicited by the first post-boundary note, full cadences elicited a centro-parietal positivity peaking at around 280 ms (see Fig. B; lateral: *F* [1, 28] = 32.241, *p* < .001; midline: *F* [1, 28] = 42.219, *p* < .001); most pronounced on midline and medial centro-posterior electrodes (Laterality × Cadence: *F* [1, 28] = 24.197, *p* < .001; medial: *F* [1, 28] = 10.166, *p* = .004; AntPost × Lat × Cadence: *F* [2, 56] = 7.651, *p* = .004; medial, central: *F* [1, 28] = 45.417, *p*< .001, medial, posterior: *F* [1, 28] = 41.966, *p* < .001), as well as on left frontal medial channels (AntPost × Lat × Hemi × Cadence: *F* [2, 56] = 3.708, *p* = .031; frontal, left, medial: *F* [1, 28] = 11.252, *p* = .002). Interestingly, this effect was connected to the acoustic effects of phrasing and the predictability (familiarity) of the phrase boundary: On the midline electrodes it was largest for Phrased (Pause × Cadence*: F* [2, 56] = 5.113, *p* = .009; Phrased: *F* [1, 28] = 6.015, *p* = .021) and No Pause (*F* [1, 28] = 28.741, *p* < .001), and within Phrased items it was most prominent when the phrase was presented for the second time (Pause × Cadence × Repetition: *F* [2, 56] = 3.166, *p* = .050 (lateral); *F* [2, 56] = 3.387, *p* = .041 (midline); Repeated, Phrased: *F* [1, 28] = 10.166, *p* = .004 (lateral); *F* [1, 28] = 10.732, *p* = .003 (midline)). Whereas for the Phrased items there appeared to be no interaction with ROI factors on non-midline electrodes, it was present for the No Pause phrases where the P3 component elicited by cadential differences was clearly largest at central medial scalp areas (AntPost × Lat × Pause × Cadence*: F*[4, 112] = 3.060, *p* = .037; No Pause, central, medial: *F*[1, 28] = 40.885, *p* < .001). In non-musicians, for repeated Phrased items, the effect of Cadence was predominantly located at Pz in the case of midline electrodes (Group × AntPost × Pause × Cadence × Repetition*: F*[4, 112] = 2.965, *p* = .023; Non-musicians, Phrased, Pz: *F*[1, 15] = 6.982, *p*= .018). This component was also largest for repeated items (Cadence × Repetition: *F*[1, 28] = 5.985, *p* = .021 (midline); Repeated: *F*[1, 28] = 28.600, *p*< .001), with this interaction at non-midline electrode sites being most pronounced at frontal (AntPost × Repetition × Cadence: *F* [2, 56] = 3.586, *p* = .048; Repeated, frontal: *F* [1, 28] = 13.621, *p* < .001), and more specifically, medial frontal sites (AntPost × Lat × Repetition × Cadence: *F* [2, 56] = 5.165, *p* = .009; Repeated, medial, frontal: *F* [1, 28] = 15.609, *p* < .001). For midline electrodes, the interaction was most pronounced at both Fz (AntPost × Cadence × Repetition: *F* [2, 56] = 6.606, *p* = .003; Repeated, Fz: *F* [1, 28] = 26.926, *p*< .001) and Cz (*F*[1, 28] = 27.805, *p*< .001). Note that for the acoustic phrasing manipulation, a P3-like response was also present as in previously reported in studies of the post-boundary music-CPS (e.g., [1]); this effect was more right lateralized (*F*[2, 56] = 11.747, *p* < .001) and had a fronto-central scalp distribution at non-midline electrode sites (frontal: *F* [2, 56] = 9.040, *p*< .001; central: *F*[2, 56] = 10.344, *p* = .001), and interacted with the factors Cadence and Repetition in similar ways as the above-described relationships.

**Fig. B.** **ERP effects of syntactic phrasing in music (i.e., strong vs. weak syntactic closure at the phrase boundary).** Baseline: −2000 to 1000 ms. Topographic maps represent the scalp distribution of the difference between Strong syntactic closure and Weak syntactic closure in the 230 to 340 ms time window. Note that only melodies with 600 ms long first post-boundary notes were used in the analysis of the effects of syntactic closure.


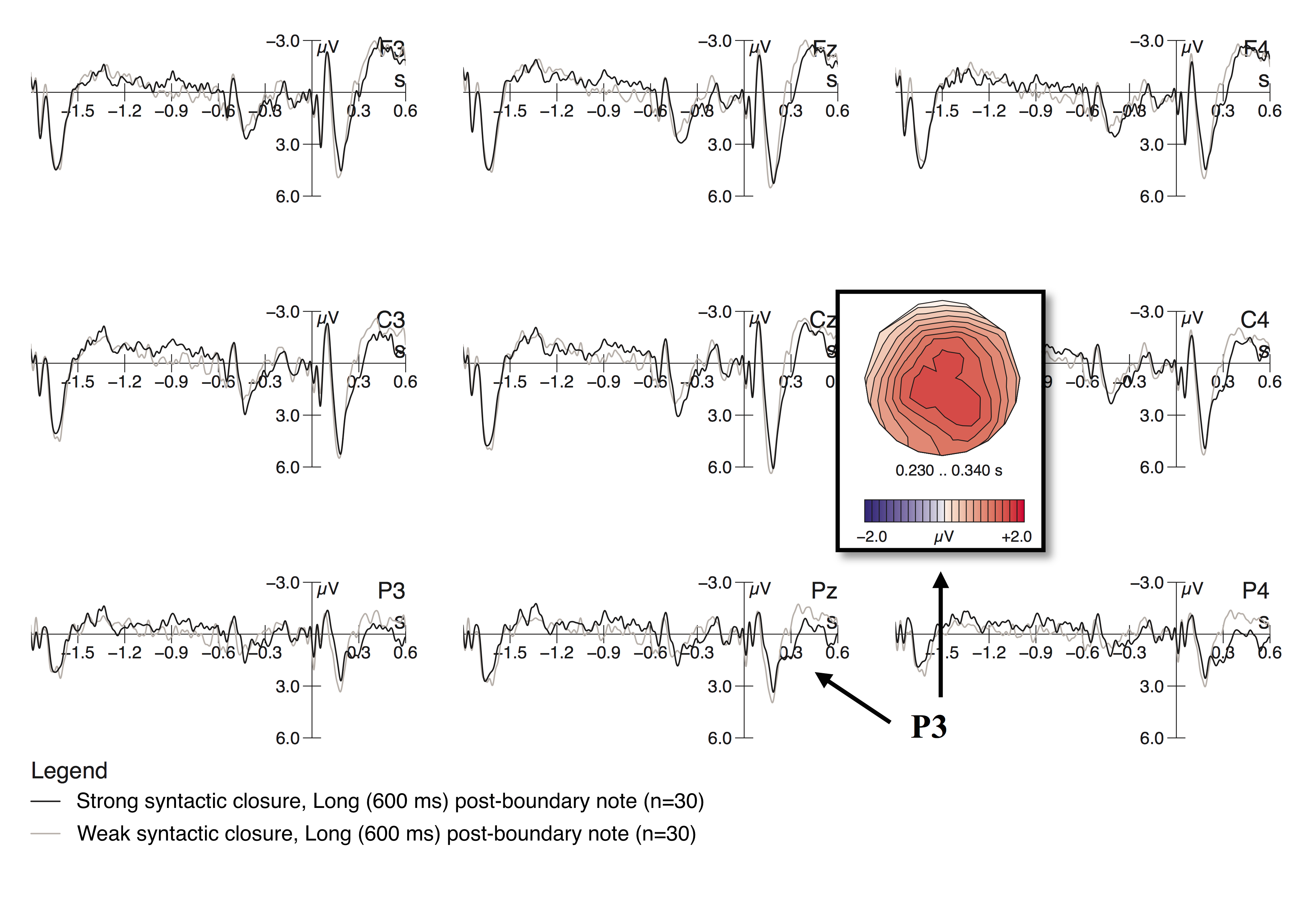


**Discussion**

Whereas we found no evidence for expectation effects (as measured by using repetition of musical phrases) to have significantly influenced either of the (local) early or late CPS-like shifts in the present study, musical syntactic cues modulated the post-boundary music-CPS. In addition, cadential cues themselves (independent of the presence of acoustic boundary cues) had an effect on neurophysiological processing of musical phrases that was independent of level of musical expertise and was reflected in the positivity peaking at around 280 ms. Similarly, Nan and colleagues [1] have previously reported a similar P280 component in non-musicians, which was modulated both by the presence of an acoustic phrase boundary and by stimulus familiarity (i.e., Chinese melodies were meant to be less familiar for German participants than German musical passages). They interpreted this effect as a novelty P3 component, reflecting ‘surprise’ elicited by the re-onset of the melody after the phrasal boundary. Interestingly, whereas the P3-like effect at the onset of the post-boundary phrase has been previously associated with item familiarity (i.e., it was largest for Chinese melodies in German non-musicians [1]), in our study, the most prominent effect in the P300 time window was seen in repeated items rather than in phrases presented for the first time. This is not surprising, since the continuation of the melody may be more expected after the end of the first and the second phrase, rather than after the third and fourth ones, whereas cultural familiarity effects might be unfolding in different ways. To sum up, the P300 complex in our study, similar to Nan and colleagues’ [1] suggestions, might represent the ‘orientation response’ associated with the re-onset of the melody after the phrase-final cues.

**References**

1. Nan Y, Knösche TR, Friederici AD. Non-musicians’ perception of phrase boundaries in music: A cross-cultural ERP study. Biol Psychol [Internet]. 2009 Sep;82(1):70–81. doi:10.1016/j.biopsycho.2009.06.002.
